# Zn(II) Curcuminate Complexes with 2,2′-bipyridine and Carboxylates

**DOI:** 10.3390/molecules24142540

**Published:** 2019-07-11

**Authors:** Sabina Grabner, Barbara Modec

**Affiliations:** Faculty of Chemistry and Chemical Technology, University of Ljubljana, Večna pot 113, 1000 Ljubljana, Slovenia

**Keywords:** curcumin, Zn(II) complexes, stability, crystal structures

## Abstract

Two novel zinc(II) compounds with curcuminate (abbreviated as cur^−^), [Zn(CH_3_COO)(cur)(bpy)](**1**)·CH_3_OH·2H_2_O (bpy = 2,2′-bipyridine) and [Zn(PhCOO)(cur)(bpy)] (**2**)·CH_3_OH, have been synthesized and characterized. Their composition has been determined by single-crystal X-ray structure analysis. Complexes **1** and **2** are similar: in both a five-fold coordination environment of zinc(II) consists of a monodentate carboxylate, a chelating bidentate 2,2′-bipyridine, and curcuminate, which is bound via a deprotonated 1,3-dione moiety. In **1**, 2,2′-bipyridine nitrogen atoms and curcuminate oxygen atoms form the base of a square pyramid, whereas the acetate oxygen occupies its apex. The O_3_N_2_ donor set in **2** defines a polyhedron which more closely resembles a trigonal bipyramid. The packing in the crystal lattices of both compounds is governed by hydrogen-bonds. Complexes **1** and **2** display higher stability than curcumin in buffered media at pH = 7.0, however, the degradation of coordinated cur^−^ is comparable to that of yellow pigment curcumin (curH) when the pH is raised to 7.2. Both complexes **1** and **2** in DMSO exhibit fluorescence with Stokes shifts of 5367 and 4634 cm^−1^, respectively.

## 1. Introduction

The Indian spice turmeric (*Curcuma Longa* L) is used in food processing (as a curry ingredient, flavor, and dye), fabric dyeing and cosmetics, and has also been used as a medical herb in Ayurvedic medicine for centuries [1]. Yellow pigment curcumin (abbreviated as curH), the major component of curcuminoids isolated from turmeric powder, is known to have a broad spectrum of medicinal benefits. Over the past five decades, numerous laboratory and clinical studies have shown the multitargeting pharmacological behavior of curH as an anticancer and anti-inflammatory agent, a promising therapeutic for neurodegenerative (e.g., Alzheimer’s disease), or metabolic diseases (e.g., diabetes, obesity), etc. [2,3,4,5,6,7,8,9].

**Scheme 1:**
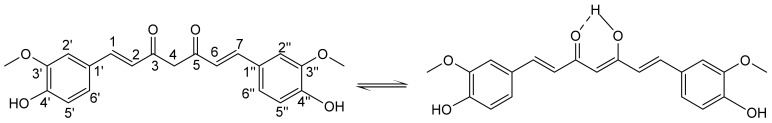
Keto-enol tautomers of curcumin with the atom numbering.

The structure of curH, ((1E,6E)-1,7-bis(4-hydroxy-3-methoxyphenyl)hepta-1,6-diene-3,5-dione), consists of two phenols with methoxy groups in the ortho position joined by a seven-carbon flexible spacer with a β-diketone moiety and two carbon–carbon double bonds [10,11]. The diketone group exhibits tautomerism with the β-diketone and two equivalent asymmetric keto-enol tautomers (Scheme 1).

NMR investigations of curH in solvents of different polarity, i.e., chloroform, methanol, acetic acid, DMSO, and DMSO/water mixtures, have shown that the keto-enol tautomer exists in solutions as the only form [12]. CurH, a weak Brønsted acid, has enolic and two phenolic protons with pKa values of 8.4, 9.9, and 10.5, respectively [13]. CurH is almost insoluble in acidic and neutral aqueous solutions. Its solubility increases at pH > 7, but already at physiological pH curcumin rapidly degrades in an autoxidation reaction to a major bicyclopentadione product [14,15,16]. CurH binding through its monoanionic enol form to metal ions is one of the possible approaches to overcome its degradation [17,18,19].

Complexation of curcuminate (cur^−^) with a plethora of metal ions ( Ru(II), Zn(II), V(IV), Cu(II), Gd(III), Co(III), Ln(III), Pt(II) or Pd(II)) not only improves its stability but also expands its possible medicinal use [18,20,21,22,23,24,25]. For example, the Ru(II) complex with η^6^-arene, curcuminato, and chlorido ligands, ([(η6-p-cymene)RuCl(cur)]), displays a good cytotoxic activity in breast MCF7 and ovarian A2780 cell lines [26]. A DNA docking study of this complex revealed the same mechanism of action as in platinum chemotherapy. The Ru(II) complex with Tröger’s base, p-cymene, and curcuminato ligands shows fast cellular uptake and displays good luminescence and cytotoxicity against cervical cancer cells [27]. Platinum(II) complexes with curcuminate can act as chemotherapeutic agents which release photoactive curcumin and an active Pt(II) species upon irradiation with visible light [28,29]. Complexes of manganese and copper with cur^−^ have been examined for their SOD activity, free radical-neutralizing abilities and antioxidant properties, e.g., [Cu(Cur)(OAc)(OH)] [30,31,32].

Zinc is attractive as a central metal ion in coordination chemistry. It is the only metal that is present in all enzyme classes, and is the second most abundant trace metal in humans after iron. In a variety of biological processes zinc performs diverse physiological functions, e.g., as a structural component (zinc fingers), as a catalytic factor (enzyme cofactors in six main enzyme classes), or as a signaling mediator [33,34]. Combinations of Zn(II) with cur^−^ and one of the aromatic spectator ligands (2,2′-bipyridine, phenanthroline, terpyridine derivatives, etc.) show antiproliferative activity in different cancer cell lines in vitro [35]. Because of the cur^−^ moiety, these complexes emit fluorescence light in the green part of the spectrum, which enables investigation of their interaction with DNA and proteins by optical methods [36,37]. Furthermore, in some Zn(II) complexes with cur^−^, anti-diabetic activity has also been observed [38].

To the best of our knowledge, only one crystal structure of a zinc(II) complex with curcuminate has been reported so far [36]. Our work has focused on Zn(II) complexes with curcuminate, 2,2′-bipyridine, and carboxylates. The variation of ligand types and their steric effect around the metal center is an essential parameter for modulating (tuning) the solubility, stability, and biological activities of metal complexes. In some studies, bipyridine derivatives have proven to be suitable spectator ligands to complete the metal ion coordination sphere along with cur^−^, and thereby improve the crystallization of the complex [36,39,40]. Acetate ions in Pt(II) complexes were shown to significantly enhance their solubility in water and chemical stability compared to the parent chlorides, while a benzoate ligand in a kiteplatin Pt(IV) analog has boosted its cytotoxicity [41,42].

**Scheme 2:**
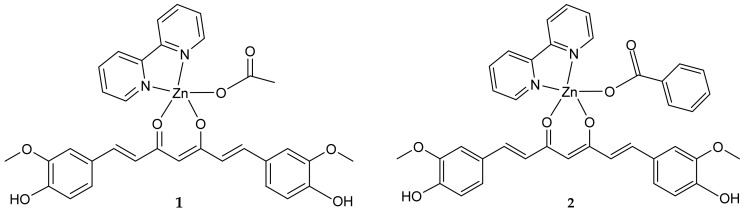
Structural formulae of [Zn(CH_3_COO)(cur)(bpy)](**1**) and [Zn(PhCOO)(cur)(bpy)](**2**).

Herein we report on the preparation, spectroscopic characterization, stability, and X-ray structures of two novel Zn(II) compounds, [Zn(CH_3_COO)(cur)(bpy)](**1**)·CH_3_OH·2H_2_O and [Zn(PhCOO)(cur)(bpy)] (**2**)·CH_3_OH (Scheme 2).

## 2. Results and Discussion

### 2.1. Crystallographic Study

**Figure 1:**
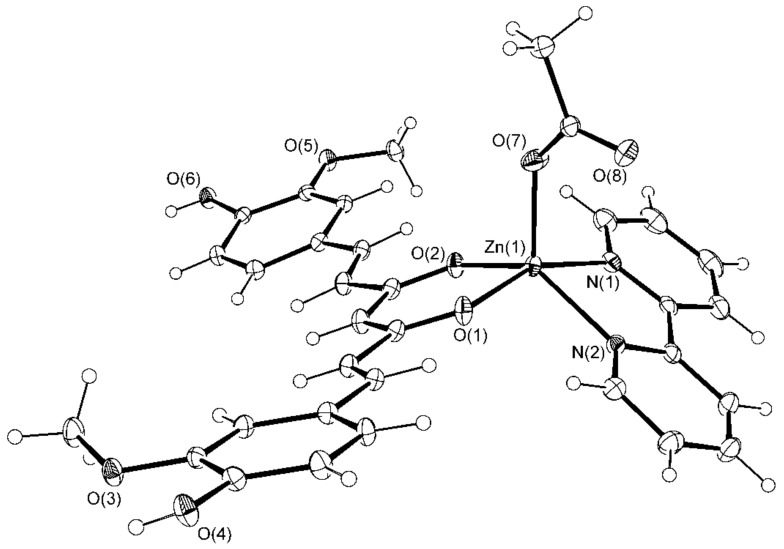
ORTEP drawing of [Zn(CH_3_COO)(cur)(bpy)](**1**) with displacement ellipsoids drawn at the 30% probability level.

**Figure 2:**
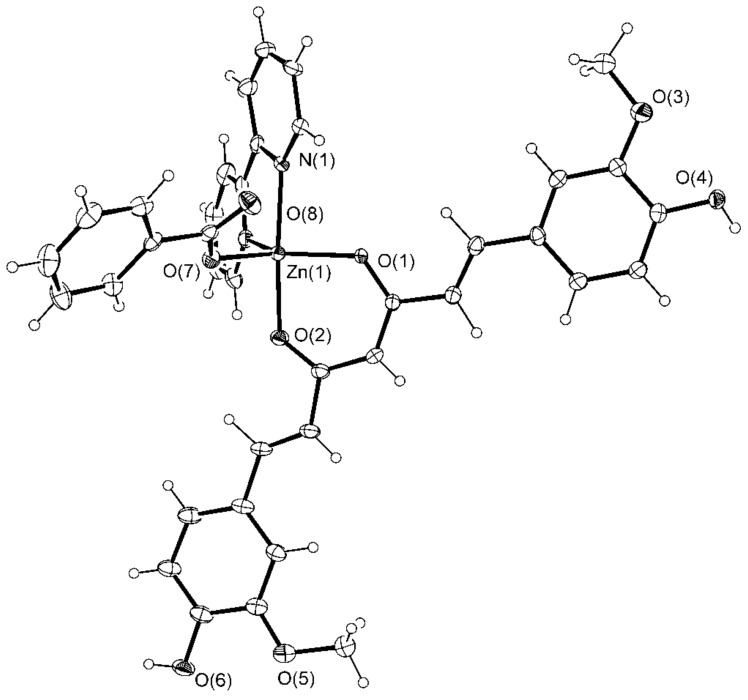
ORTEP drawing of [Zn(PhCOO)(cur)(bpy)](**2**) with displacement ellipsoids drawn at the 30% probability level.

[Zn(CH_3_COO)(cur)(bpy)](**1**)·CH_3_OH·2H_2_O crystallizes in a monoclinic space group *P* 2_1_/*a*, and [Zn(PhCOO)(cur)(bpy)](**2**)·CH_3_OH crystallizes in a monoclinic space group *C c*. Their solid-state structures consist of neutral complex molecules and solvent molecules of crystallization. The complex molecules of both compounds are similar. Their ORTEP drawings are shown in Figure 1 and Figure 2, whereas relevant geometric parameters are listed in Table 1.

The five-fold coordination environment of zinc(II) in [Zn(CH_3_COO)(cur)(bpy)](**1**) consists of a monodentate acetate and two chelating bidentate ligands, 2,2′-bipyridine as a N,N-donor and monoanionic curcuminate as an O,O-donor. The latter is a deprotonated enolic form of curcumin. With the coordination of its central O,O-moiety to zinc(II), a six-membered chelate ring forms. The O_3_N_2_ donor set defines a polyhedron, which more closely resembles a distorted square pyramid than a trigonal bipyramid. The *τ* parameter amounts to 0.41. For an ideal tetragonal geometry, *τ* equals zero, whereas it becomes unity in the case of a perfect trigonal bipyramidal geometry [43].

**Figure 3:**
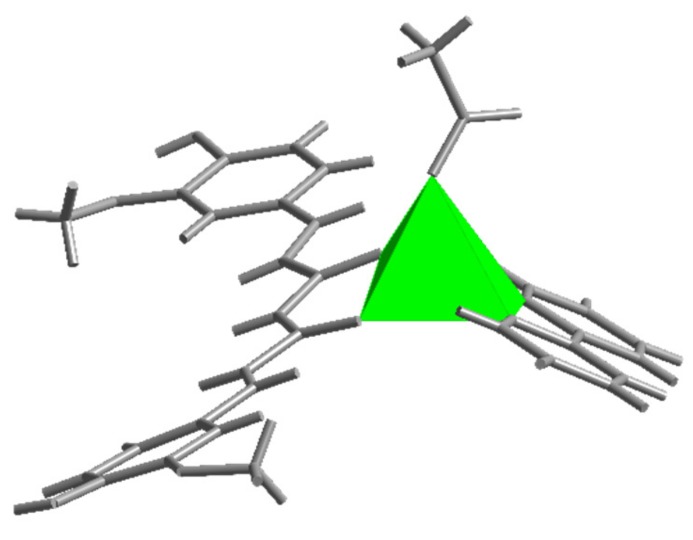
The square pyramidal environment of zinc ion in [Zn(CH_3_COO)(cur)(bpy)](**1**).

**Figure 4:**
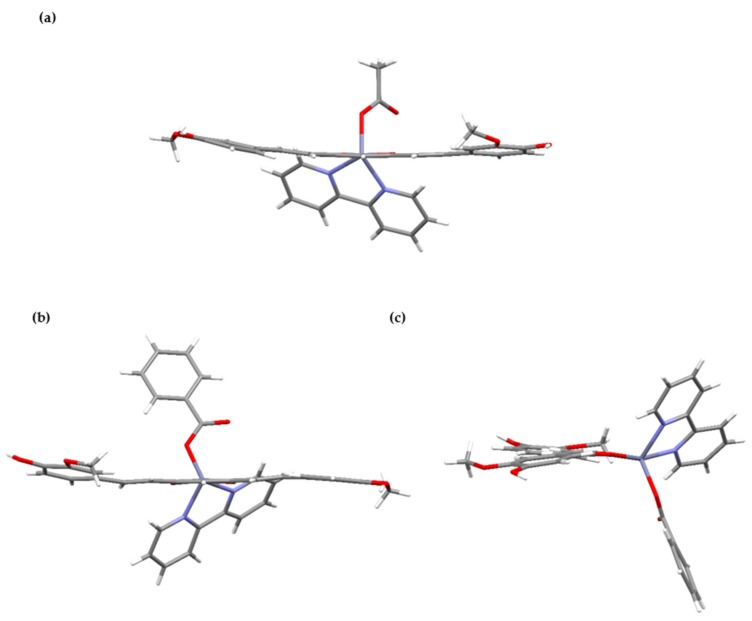
Projections of complex molecules [Zn(CH_3_COO)(cur)(bpy)](**1)** (**a**) and [Zn(PhCOO)(cur)(bpy)](**2**) (**b**,**c**). The top drawing shows a slight curvature of the cur^−^ ligand in **1**, whereas a twisted orientation of the aromatic planes in **2** is shown by the bottom ones.

2,2′-bipyridine nitrogen atoms and curcuminate oxygen atoms define the base of the square pyramid whose apex is occupied with the acetate oxygen (Figure 3). The curcuminate-to-zinc bond lengths are 2.001(1) and 2.016(1) Å, the 2,2′-bipyridine-to-zinc bond lengths are 2.123(1) and 2.162(2) Å, and the carboxylate binds at 1.973(1) Å. The other carboxylate oxygen, O(8), is at 3.303(2) Å, a distance which no longer suggests a significant bonding interaction. The cur^−^ ligand is slightly curved (Figure 4), and its chain backbone is almost planar with the phenyl rings deviating from the plane. A dihedral angle of 16.75(9)° is formed between the planes of the phenyl rings. The orientations of the phenyl rings are such that their methoxy substituents are located on the opposite sides of the conjugated chain system. Within one aromatic ring, an intramolecular hydrogen-bond between its OH and methoxy entities may be observed.

**Figure 5:**
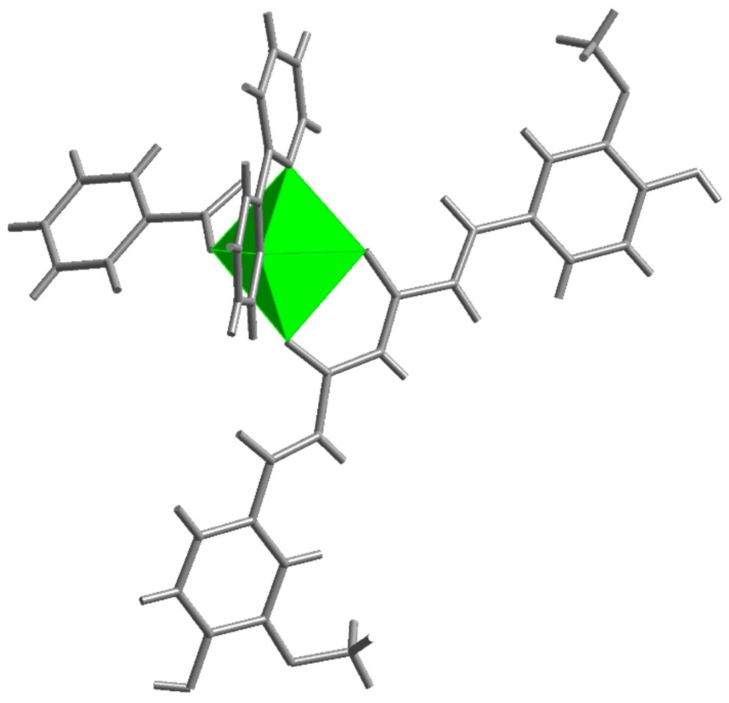
The trigonal bipyramidal environment of zinc ion in [Zn(PhCOO)(cur)(bpy)](**2**).

Benzoate, the carboxylate anion in [(Zn(PhCOO)(cur)(bpy))](**2**), is bound in the same way as acetate in **1**. The coordination environment of the metal in **2** more closely resembles a trigonal bipyramid, as shown by the value of the *τ* parameter, 0.63 (Figure 5). The 2,2′-bipyridine ligand is coordinated in a chelating manner along one edge of the pyramid, and its nitrogen atoms occupy one equatorial and one apical site (Figure 5). The cur^−^ ligand, coordinated in a similar manner, spans a different edge of the pyramid with one donor atom located at the equatorial site and the other at the opposite apex. The carboxylate oxygen occupies the remaining equatorial site. The bonding parameters in **2** are similar to those determined for the acetate compound: The curcuminate-to-zinc bond lengths are 2.007(3) and 2.051(3) Å, the 2,2′-bipyridine-to-zinc bond lengths are 2.112(4) and 2.121(4) Å, and the carboxylate binds at 1.987(3) Å. Surprisingly, the distance between the non-coordinated carboxylate oxygen and zinc(II) ion is shorter than in **1**, i.e., 3.127(3) vs. 3.303(2) Å, respectively. The cur^−^ ligand of **2** deviates from planarity. Its conformation may be described as twisted (Figure 4). An angle of 11.3(2)° is formed between the planes of the phenyl rings. As in **1**, the methoxy groups are in the trans position with respect to the conjugated chain, with neither being engaged in intramolecular hydrogen-bonding with an adjacent OH group.

The composition of the only structurally characterized zinc(II) complex with curcuminate, [ZnCl(cur)(bpy-9)], resembles that of the title compounds: It is a complex with a derivative of 2,2′-bipyridine, i.e., a dinonyl-2,2′-bipyridine, with a chloride in place of a monodentate monoanionic ligand [36]. In [ZnCl(cur)(bpy-9)], zinc(II) features a distorted square pyramidal environment with chloride occupying its apex. The curcuminate-to-zinc distances, i.e., 2.016(2) and 2.031(2) Å, are comparable to those in **1** and **2**. Moreover, the comparison extends to the bite angle spanned by the curcuminate donor atoms, 90.0(1)° for [ZnCl(cur)(bpy-9)] vs. 92.22(5)° for **1** and 89.75(11)° for **2**. The geometries of **1** and **2** find a close comparison to the complexes with acetylacetonate (acac^−^), a smaller ligand that may be considered as a prototype of the curcuminate. For instance, in [Zn(bpy)(acac)_2_], with a N_2_O_4_ donor set defining a distorted octahedron, the corresponding bond lengths are 2.042(1)–2.089(1) Å and the acetylacetonate bite angles are 88.44(6) and 88.78(6)° [44].

In a dinuclear [Zn_2_(tpy)(acac)_4_] with a O_4_N donor set defining a trigonal bipyramidal environment, the acac-to-zinc bond lengths span a wider interval, 1.966(2)–2.089(2) Å, whereas the bite angles are 88.39(6)–90.42(8)° [45]. In all known structures of the curcuminate metal complexes, the ligand adopts a chelating bidentate O,O-donor coordination mode with the curcuminate-to-metal bonds depending upon the nature of the metal ion [21,22,26,36,39,46,47,48,49,50,51]. For example, the La–O bond distances involving the cur^−^ ligand in [La(R-tpy)(cur)(NO_3_)_2_] (R-tpy = 4’-phenyl-2,2′:6’,2″-terpyridine) are as long as 2.400(4) and 2.410(4) Å [22]. In [VO(cur)(phen)Cl], the corresponding bond lengths are slightly shorter than in the zinc complexes, i.e., 1.959(4) and 1.973(5) Å [21]. The shortest bonds, 1.878(4) and 1.880(4) Å, were determined for a cobalt(III) complex with the [Co(cur)L] (L = a tetradentate phenolate-based ligand) composition [49]. A close inspection of the structures reveals that the curcuminate ligand typically shows at least a slight deviation from planarity and that a pair of methoxy groups can be positioned either on the opposite sides of the chain backbone of the ligand, as observed in **1** and **2**, or on the same side, as demonstrated by [VO(cur)(phen)Cl] [21]. In some complexes, the hydroxyl group forms an intramolecular hydrogen-bond with the adjacent methoxy group. The latter probably depends upon the involvement of the hydroxyl moiety in other intermolecular interactions. The complex molecules of **1** and **2** possess functional groups capable of hydrogen-bonding: hydroxyl groups, which can serve both as the acceptors and donors of hydrogen-bonds, and methoxy oxygens and non-coordinated carboxylate oxygens, which can serve only as the acceptors. In addition, the title compounds contain solvent molecules of crystallization, methanol, and water, which also participate in hydrogen-bonding. The packing in the crystal lattices of both compounds is thus governed by hydrogen-bonds. As expected, all good hydrogen-bond donors and acceptors are involved in interactions [52]. In [Zn(PhCOO)(cur)(bpy)](**2**)·CH_3_OH, each complex molecule makes four hydrogen-bonding interactions: Both hydroxyl groups, a non-coordinated benzoate oxygen, and a coordinating oxygen of the cur^−^ ligand are involved (Table 2).

**Figure 6:**
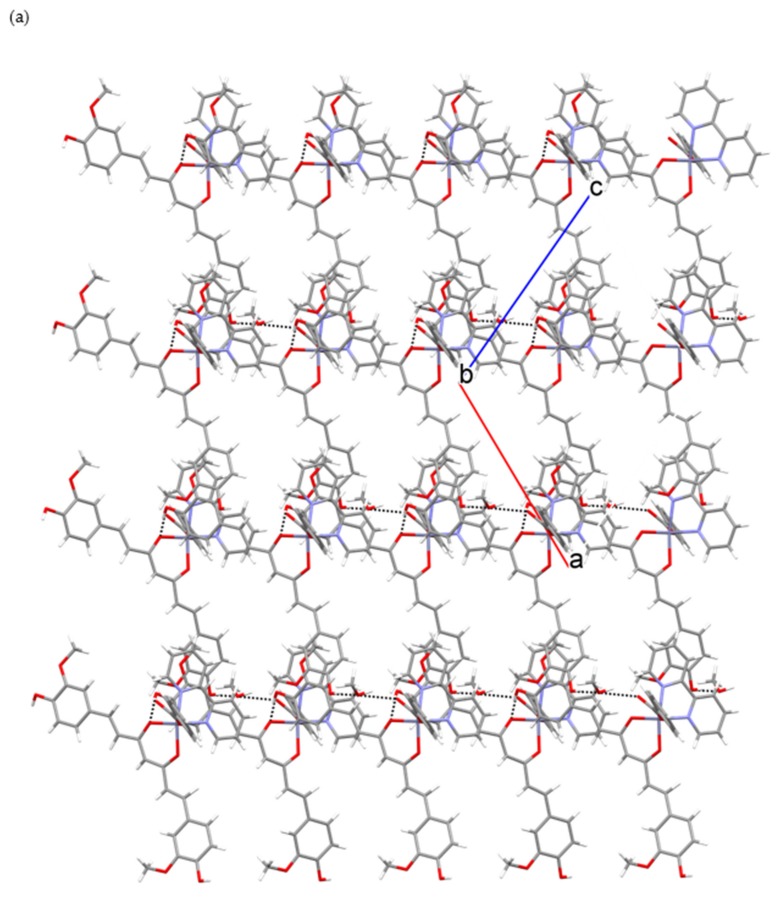

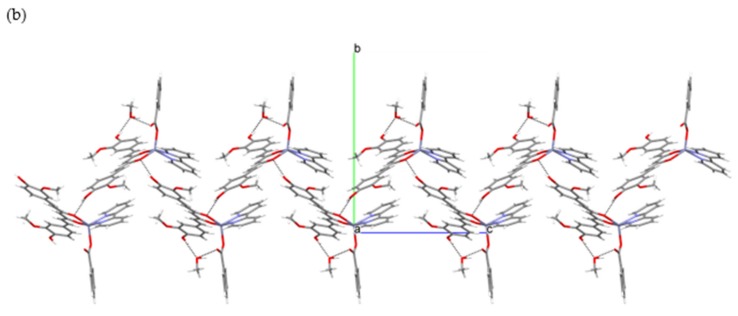
Hydrogen-bonding in [Zn(PhCOO)(cur)(bpy)](**2**)·CH_3_OH: (**a**) Section of a layer of hydrogen-bonded [Zn(PhCOO)(cur)(bpy)](**2**) and methanol molecules. (**b**) A view of the layer along *a* axis.

One OH moiety is hydrogen-bonded to a methanol molecule, whereas the other interacts with the dione oxygen. The free benzoate oxygen makes a hydrogen-bonding interaction with the methanol molecule. As a result, each complex molecule is hydrogen-bonded to two adjacent methanols and two complex molecules, producing an infinite two-dimensional array (Figure 6). The layers that are corrugated stack along the *b*-axis. The packing of complex molecules is such that it allows π–π stacking interactions of 2,2′-bipyridine and curcuminate phenyl rings (centroid–centroid distance = 3.577(3) Å, interplanar distance = 3.428(2) Å, dihedral angle = 2.3(2)°, offset angle = 16.6°) [54].

**Figure 7:**
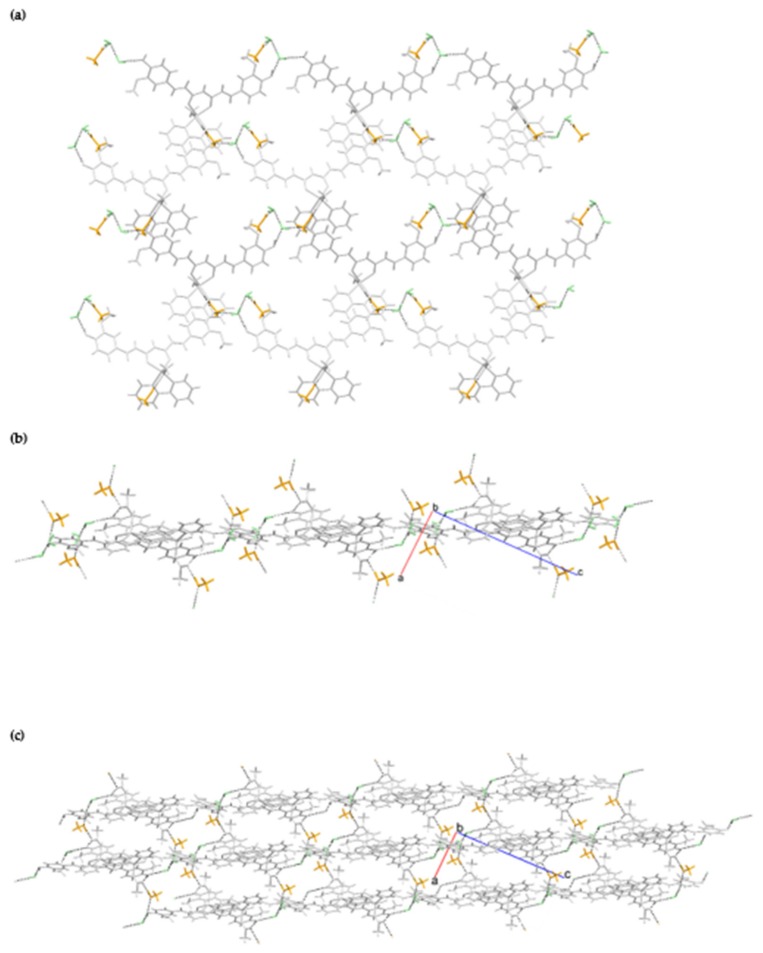
Hydrogen-bonding in [Zn(CH_3_COO)(cur)(bpy)](**1**)·CH_3_OH·2H_2_O: (**a**) A perpendicular view of the layer. (**b**) A view along the layer. (**c**) Stacking of layers with the agency of methanol linkers. Color code: light grey = [Zn(CH_3_COO)(cur)(bpy)](**1**) molecules, orange = methanol, and green = water molecules.

The presence of another structural element capable of hydrogen-bonding in [Zn(CH_3_COO)(cur)(bpy)](**1**)·CH_3_OH·2H_2_O, i.e., water molecules of crystallization, leads to a more complicated pattern of connectivity. Again, all good hydrogen-bond donors are involved (Table 3). One of the two hydroxyl moieties is engaged in an intermolecular interaction with a free carboxylate oxygen, whereas the other is hydrogen-bonded to a water molecule. The shortest O∙∙∙O contact involves the hydroxyl and the carboxylate. The [Zn(CH_3_COO)(cur)(bpy)](**1**) and water molecules are linked into layers with methanol molecules attached to their surfaces (Figure 7). Methanol molecules serve as linkers between the layers. The π–π stacking interactions between 2,2′-bipyridine rings may also be observed (centroid–centroid distance = 3.815(1) Å, interplanar distance = 3.4214(8) Å, dihedral angle = 0.95(9)°, offset angle = 26.3°) [54].

### 2.2. Synthesis

Novel compounds of Zn(II), **1**·CH_3_OH·2H_2_O, and **2**·CH_3_OH were prepared by a direct one-pot reaction of Zn(II) aliphatic or aromatic carboxylates with 2,2′-bipyridine and curcuminate. Deprotonation of curH was carried out in methanol by slow addition of potassium hydrogen carbonate at room temperature. Syntheses were conducted in methanol at 45 °C. The mixtures were heated for approximately 90 min. Crystals were obtained from the corresponding filtrates after a few days at 5 °C.

### 2.3. Solubility and Stability

**Figure 8:**
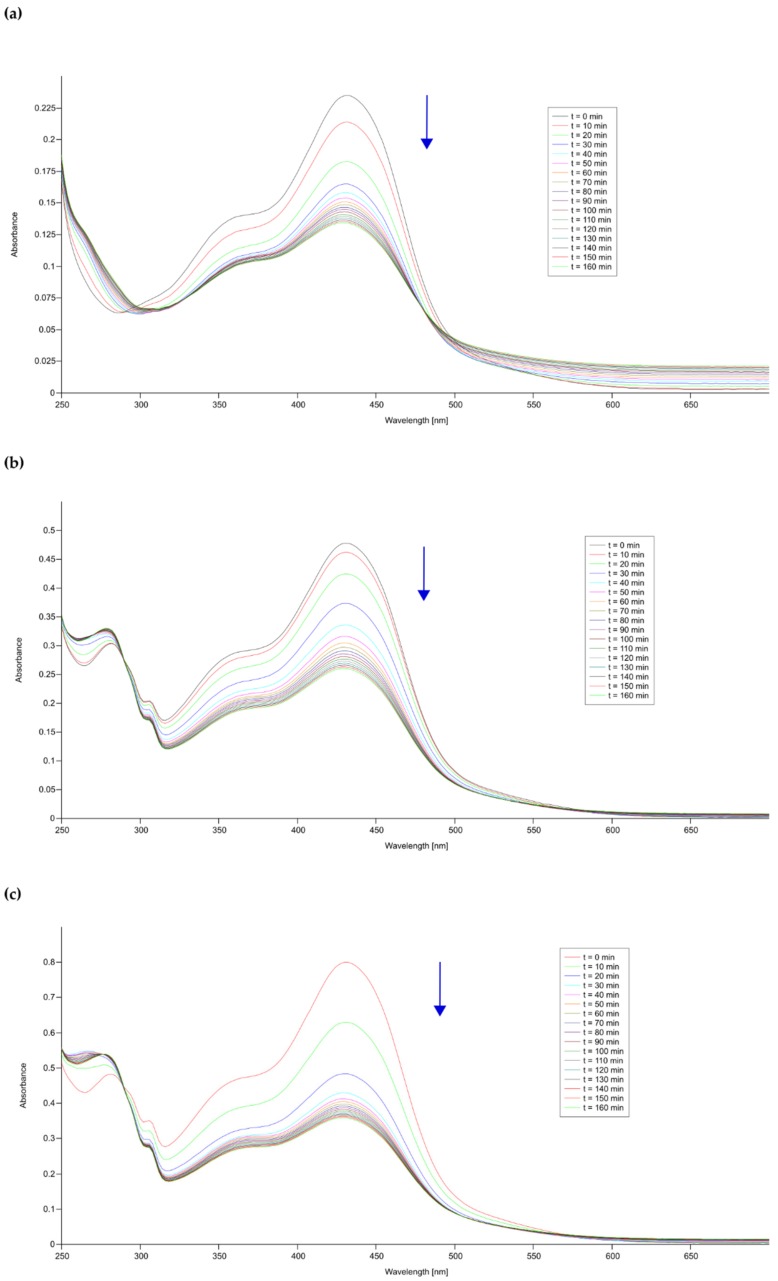
Time-dependent UV-vis spectra of (**a**) curH, (**b**) **1**, and (**c**) **2** in 90 vol.% phosphate buffered saline (PBS, pH = 7.2) + 10 vol.% DMSO solutions.

**Figure 9:**
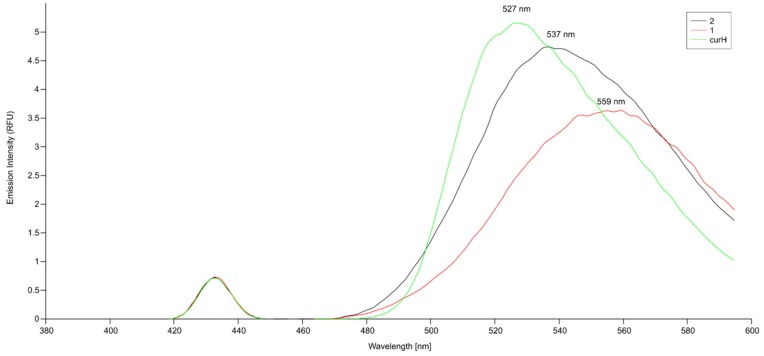
Emission spectra of 0.1 µM curH, **1**, and **2** in DMSO (λ_ex_ = 435 nm for curH; λ_ex_ = 431 nm for **1** and **2**).

The title compounds are poorly soluble in DMSO, DMF, methanol, and almost insoluble in water, dichloromethane, acetone, chloroform, acetonitrile, and ethyl acetate. The stability of curH, **1** and **2** in DMSO, 90 vol.% 100 mM NaCl + 10 vol.% DMSO, and under pseudo-physiological conditions (90 vol.% PBS (phosphate buffered saline) + 10 vol.% DMSO) were monitored for 160 min and also after 24 h in DMSO and 90 vol.% 100 mM NaCl + 10 vol.% DMSO by UV-vis spectroscopy. In the 420 to 460 nm spectral range, the DMSO solutions of complexes **1** and **2** showed two absorption bands at 431 and 452 nm, while curH had only one broad band at 436 nm. For all, the strong band at 431 nm could be assigned to a π–π* transition [55]. When the solvent was replaced from DMSO to aqueous buffered media with 10 vol.% DMSO or 100 mM NaCl with 10 vol.% DMSO, the position of the π–π* transition remained at almost the same wavelength. In DMSO, the absorption bands at 430 and 452 nm for **1** and **2** and at 436 nm for curH retained their intensities for over 24 h (Figures S1–S3). In 90 vol.% 100 mM NaCl + 10 vol.% DMSO, curH, **1**, and **2** were stable in the first 160 min, but after 24 h absorbance of the maximum band dropped by 52, 50, and 60%, respectively (Figures S4–S6). On the contrary, in buffered aqueous solutions at pH = 7.2 (Figure 8) the absorption band at 431 nm for curH, **1**, and **2** diminished quickly within 160 min by 44, 46, and 55%, respectively. At pH = 7.0, the decompositions of curH, **1**, and **2** were slower, and after 160 min their concentrations dropped by 36, 26, and 29%, respectively (Figures S7–S9). Our results confirm the good stability of curH, **1**, and **2** in aprotic solvents. In buffered solutions with pH between 7.2 and 7.4, **1** and **2** decompose as fast as curH. For comparison, in the absorption spectra of similar Zn(II) complexes with cur^−^, 2,2′-bipyridine derivatives, and chlorido ligands recorded under physiological pH, absorption maxima in the range of 408–450 nm drop only by ca. 25% in 24 h and by ca. 35% in 48 h [36]. The emission spectra of 1 µM solutions of curH, **1**, and **2** in DMSO obtained upon excitation with 430 nm light show 4013, 5367, and 4634 cm^−1^ Stokes shifts, respectively (Figure 9). The fluorescence intensity of complex **2** is comparable with that of curH, while the intensity of **1** is considerably lower.

### 2.4. Spectroscopic Studies

**Figure 10:**
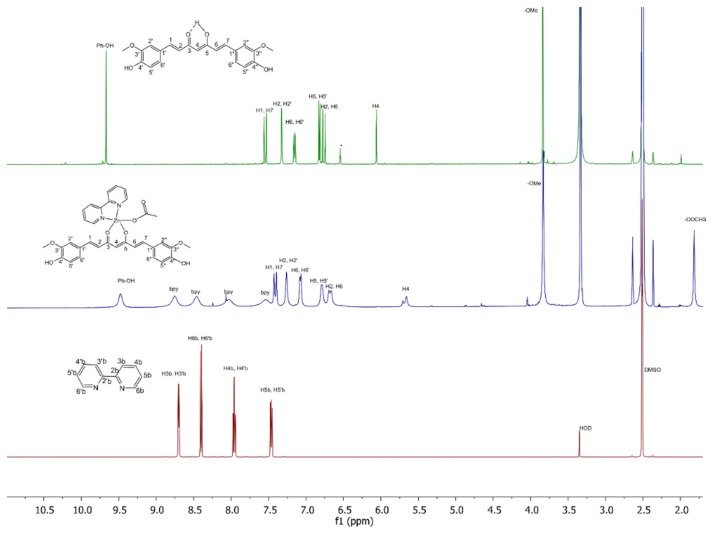
^1^H NMR (500 MHz) spectra of yellow pigment curcumin (curH, top), complex **1** (middle), and bpy (bottom) in DMSO-*d_6_*.

The ^1^H NMR spectra of the title compounds in DMSO-*d_6_* show signals for protons of bound ligands, and the values of their integrals are consistent with the presence of curcuminato, 2,2′-bipyridine and acetato (in **1**)/benzoato (in **2**) ligands in a 1:1:1 ratio (Figures S10 and S11). Peaks were assigned using COSY spectra of **1** and **2** in DMSO-*d_6_* and ^1^H NMR spectra of the Zn(II) starting compound in DMSO-*d_6_* (Figure 10, Figures S12–S14) and are listed in the experimental section. The resonances belonging to cur^−^ were seen, although they were somewhat broadened. They are shifted slightly upfield compared to curH (Δδ from 0.19 to 0.03 ppm for **1** and **2**). The most substantial change upon coordination of cur^−^ to Zn(II) ion is observed for methylene H-4 which is located closest to the metal ion. Its resonance is shifted upfield by 0.4 ppm. The resonances of 2,2’-bipyridine are broad, probably due to chemical exchange processes or intra- or intermolecular interactions, and therefore cannot be interpreted precisely [56]. They appear in the 9.1–7.3 ppm range. All four are shifted downfield (approximately Δδ = 0.07 ppm) compared to the free 2,2′-bipyridine, and have no cross peaks in the COSY spectra. NMR spectra (^13^C NMR, (^1^H,^13^C) HMQC), which could further assist in the assignment process, could not be recorded due to the poor solubility of compounds in DMSO, as well as in other solvents.

The FTIR spectra of curH and the title compounds are presented in Figures S15–S17 and the absorption bands are listed in the experimental section. The assignment of bands in the spectrum of curH follows the literature report for the β-keto-enol tautomer [57,58]. Phenolic ν(OH) vibrations absorb at 3510 cm^−1^. Strong bands at 1626 and 1601 cm^−1^ have mixed ν(C=O) and ν(C=C) character. The most intense band in the spectrum of curH at 1500 cm^−1^ belongs to mixed vibrations (ν(C=O), δ(CC=O), and δ(CC^1^C)). Bands at 1458 and 1427 cm^−1^ have their origin in deformation vibrations of the two methyl groups. Intense bands between 1277 and 1180 cm^−1^ are attributed to the in-plane deformation vibration of phenyl rings and skeletal in-plane deformations. However, for both compounds the assignment of cur^−^ bands could be inaccurate due to the overlay with the byp and acetate/benzoate absorptions (Figure S18). The latter also hampers identification of the carboxylate-binding mode in their infrared spectra, which is possible for simpler complexes from the positions of the ν_as_(COO) and ν_s_(COO) absorptions [59,60].

## 3. Materials and Methods

### 3.1. General

The commercial reagents were Aldrich or Fluka reagent-grade products. Curcumin was synthesized according to the published procedure [61]. PBS was prepared following a published procedure [62].

### 3.2. Measurements

Elemental C, H, N analyses were carried out on a Perkin-Elmer CHN Analyzer 2400. ATR-IR spectra were measured on a Perkin Elmer Spectrum 100 Fourier Transform Infrared Spectrometer using a diamond crystal plate in the range of 4000–600 cm^−1^ (PerkinElmer Ltd., Beaconsfield, Buckinghamshire, UK). UV-vis absorbance spectra were recorded at 25 °C on a Perkin-Elmer LAMBDA 750 UV/VIS spectrometer with 18 cycle measurements in a quartz cuvette with a 1 cm path length in the 250–700 nm range. Each cycle lasted 600 s. Stock DMSO solutions of curH, **1**, and **2** were prepared with molar concentrations in the 0.1–0.2 mM range. The stock solution was diluted ten times with DMSO or PBS buffer just before the measurement. Fluorescence measurements were carried out on a Perkin-Elmer LS 55 Fluorescence Spectrometer (PerkinElmer Ltd., Beaconsfield, Buckinghamshire, UK).in a Perkin Elmer Quartz SUPRASIL cuvette (10 mm path length). Solutions of curH, **1**, and **2** in DMSO were prepared with molar concentrations in the 10–0.1 µM range. The bandwidths for excitation and emission were 10 nm.

Thermogravimetric analyses (TGA) were run on a Mettler-Toledo TGA/DSC1 instrument (Mettler-Toledo, GmbH, Greifensee, Switzerland) in an air atmosphere with a purge rate of 100 mL min^−1^. The samples were heated at 10 °K/min in a temperature range from 25 to 1000 °C. A sample size of around 5 mg was put in 150 µL alumina crucible. The TGA curves were corrected by running an automatic blank curve subtraction. ^1^H NMR spectra were recorded on a Bruker Avance III 500 spectrometer (Bruker Corporation, Billerica, MA, USA) with standard pulse programs at 500.10 MHz (^1^H). 2D (^1^H,^1^H) COSY spectra were recorded in a gradient-enhanced mode on a Bruker Avance III 500 spectrometer with standard pulse programs at 500.10 MHz (^1^H). The NMR measurements were performed in DMSO-*d_6_* at 302 °K with TMS as an internal standard.

### 3.3. Syntheses

#### 3.3.1. Synthesis of [Zn(PhCOO)_2_]

A suspension of ZnO (1.00 g, 12.3 mmol) in 10 mL of distilled water was treated with benzoic acid (3.60 g, 29.5 mmol) and dissolved in 10 mL of methanol at the temperature of 50 °C. An additional 1.20 g of benzoic acid in 100 mL methanol was added until the solution became clear. After slow evaporation of the colorless solution at room temperature for three days, the white crystalline product was filtered off and washed with several portions of diethyl ether. The yield was 45%. Anal. C 54.79, H 2.97%, calcd for C_14_H_10_O_4_Zn, C 54.77, H 3.28%. IR, ν (cm^−1^): 1632 (s), 1595 (vs), 1575 (vs), 1540 (vs), 1527 (s), 1494 (s), 1407 (vs), 1312 (m), 1175 (s), 1158 (vs), 1001 (vs), 1070 (vs) 1026 (b), 865 (vs), 841 (vs), 818(vs), 713 (s), 694(s), 684 (vs), 676 (m). ^1^H NMR (500 MHz), δ/ppm (DMSO-d_6_): 7.98–7.92 (m, 2H, a), 7.52–7.45 (m, 1H, c), 7.44–7.38 (m, 2H, b). TGA anal. Zn 21.19% calcd. for C_33_H_34_N_2_O_10_Zn, Zn 21.26%.

#### 3.3.2. Synthesis of [Zn(CH_3_COO)(cur)(bpy)](**1**)·CH_3_OH·2H_2_O

A solution of 2,2′-bipyridine (79.4 mg, 0.508 mmol) in 2 mL of methanol was added dropwise to a stirred 5 mL solution of [Zn(CH_3_CO_2_)_2_]·2H_2_O (118.2 mg, 0.538 mmol). Crushed KHCO_3_ (41.2 mg, 0.412 mmol) was slowly added to a stirred suspension of curH (188 mg, 0.510 mmol) in 30 mL of methanol. The mixture was left stirring until the solution became red. Afterwards, the solution of cur^−^ was added dropwise. The reaction mixture was stirred at 45 °C for an hour. Afterwards, the solution was filtered and the resulting filtrate was left in an open beaker at 5 °C. Within a few days, red-orange crystals of **1**·CH_3_OH·2H_2_O formed. The yield was 30%. Note: The crystals became opaque when taken out from the mother liquor. Based on the NMR spectrum, TGA and elemental analyses, the crystals gave off weakly bound methanol. Anal. C 58.01, H 4.91, N 4.05%, calcd for C_33_H_34_N_2_O_10_Zn, C 57.94, H 5.01, N 4.10%. IR, ν (cm^−1^): 3600 – 3000 (vvb), 1622 (m), 1597 (m), 1579 (w), 1531 (s), 1504 (vvs), 1403 (vs), 1279 (vs), 1242 (s), 1216 (s), 1156 (vs), 1123 (vs), 1057 (w), 980 (vs), 966 (vs), 850 (s), 811(s), 760 (vs), 733 (s), 665 (w), 652 (m), 628 (w), 607 (m). ^1^H NMR (500 MHz), δ/ppm (DMSO-*d_6_*): 9.48 (s, 2H, -OH), 8.75 ( br s, 2H, bpy), 8.46 ( br s, 2H, bpy), 8.02 ( br s, 2H, bpy), 7.54 ( br s, 2H, bpy), 7.41 (d, *J* = 15.5 Hz, 2H, H-1, H-7), 7.26 (br s, 2H, H-2″,H-2″), 7.08 (d, *J* = 8.2 Hz, 2H, H-6″,H-6″), 6.79 (d, *J* = 4.5 Hz, 2H, H-5′,H-5″), 6.68 (d, *J* = 15.5 Hz, 2H, H-2,H-6), 5.70 (s, 1H, H-4), 3.83 (s, 6H, -OCH_3_), 1.82 (s, 3H, -OOCH_3_). TGA anal. Zn 9.18% calcd. for C_33_H_34_N_2_O_10_Zn, Zn 9.56%.

#### 3.3.3. Synthesis of [Zn(PhCOO)(cur)(bpy)](**2**) CH_3_OH

A solution of 2,2′-bipyridine (79.4 mg, 0.508 mmol) in 2 mL of methanol was added dropwise to a stirred 5 mL solution of [Zn(PhCOO)_2_] (156.4 mg, 0.508 mmol). Crushed KHCO_3_ (41.2 mg, 0.412 mmol) was slowly added to a stirred suspension of curH (188 mg, 0.510 mmol) in 30 mL of methanol. The mixture was left stirring until the solution became red. The solution of cur^−^ was added dropwise to the stirred solution of the Zn(II) starting material and 2,2′-bipyridine at 45 °C. After one hour, the solution was filtered. The filtrate was left in an open beaker at 5 °C. Yellow-orange crystals of **2**·CH_3_OH formed within few days. The yield was 35%. Note: The crystals became opaque when taken out from the mother liquor. Based on the NMR spectrum, TGA and elemental analyses, the crystals gave off weakly bound methanol. Anal. C 63.86, H 4.46, N 3.92%, calcd for C_38_H_32_N_2_O_8_Zn, C 64.28, H 4.54, N 3.95%. IR, ν (cm^−1^): 3200–3000 (vvb), 1622 (m), 1594 (m), 1562 (w), 1537 (m), 1503 (vvs), 1471 (w), 1462 (w), 1444 (w), 1403 (s), 1379 (vs), 1311 (w), 1279 (vs), 1254 (s), 1216 (m), 1187 (w), 1158 (vs), 1122 (vs), 1060 (w), 1025 (vs), 984 (vs), 969 (vs), 861 (w), 846 (m), 815 (m), 758 (vs), 732 (m), 719 (vs), 691 (w), 677 (m), 652 (m), 629 (w), 609 (w). ^1^H NMR (500 MHz), δ/ppm (DMSO-*d_6_*): 9.47 (s, 2H, –OH), 9.00–7.95 (m, 8H, bpy), 7.93 (d, *J* = 7.6 Hz, 2H, a (PhCOO^−^) ), 7.36–7.5 (m, 5H, H-1, and H-7 (cur^−^), b and c (PhCOO^−^)), 7.27 (br s, 2H, H-2′,H-2″), 7.08 (d, *J* = 8.4 Hz, 2H, H-6′,H-6″), 6.79 (d, *J* = 8.4 Hz, 2H, H-5′,H-5″), 6.69 (d, *J* = 15.7 Hz, 2H, H-2,H-6), 5.70 (s, 1H, H-4), 3.83 (s, 6H, -OCH_3_). TGA anal. Zn 9.21% calcd. for C_38_H_32_N_2_O_8_Zn, Zn 9.21%.

### 3.4. X-Ray Structure Determinations

Data were collected on an Agilent SuperNova dual source diffractometer (Agilent Technologies XRD Products, Oxfordshire, UK) with an Atlas detector at 150 °K using Mo-K*α* radiation (*λ* = 0.71073 Å). The data were processed using CrysAlis PRO software [63]. The coordinates of the majority of non-hydrogen atoms were found via direct methods using the structure solution program SHELXS [64]. The positions of the remaining non-hydrogen atoms were located by use of a combination of least-squares refinement and difference Fourier maps in the SHELXL 97 program [64]. Figures depicting the structures were prepared by Ortep3, CrystalMaker® X and Mercury [65,66,67].

Crystal data for **1**, C_34_H_38_N_2_O_11_Zn (716.03 g mol^−1^): monoclinic *P* 2_1_/*a*, *a* = 8.79160(13) Å, *b* = 20.8829(3) Å, *c* = 17.7951(2) Å, *β* = 92.5369(13)°, *V* = 3263.88(8) Å^3^, *Z* = 4, ρ_calcd_ = 1.457 g cm^−3^, *μ* = 0.818 mm^−1^; 24502 reflections collected (8775 independent, *R*_int_ = 0.0285). The final residues were *R* = 0.0371, and *wR*_2_ = 0.0817 (7011 reflections, *I* > 2*σ*(*I*)), and *R* = 0.0532, and *wR*_2_ = 0.0894 (all data).

Crystal data for **2**, C_39_H_36_N_2_O_9_Zn (742.07 g mol^−1^): monoclinic *C c*, *a* = 13.8388(5) Å, *b* = 18.3164(6) Å, *c* = 14.6960(5) Å, *β* = 114.141(4)°, *V* = 3399.3(2) Å^3^, *Z* = 4, ρ_calcd_ = 1.450 g cm^−3^, *μ* = 0.784 mm^−1^; 9986 reflections collected (6067 independent, *R*_int_ = 0.0281). The final residues were *R* = 0.0425, and *wR*_2_ = 0.1000 (5225 reflections, *I* > 2*σ*(*I*)), and *R* = 0.0550, and *wR*_2_ = 0.1073 (all data).

CCDC-1909739 (**1**) and -1909738 (**2**) contain the supplementary crystallographic data for this paper. These data can be obtained free of charge at http://www.ccdc.cam.ac.uk/conts/retrieving.html (or from the Cambridge Crystallographic Data Centre, 12 Union Road, Cambridge CB2 1EZ, UK; fax: +44 1223 336033).

## 4. Conclusions

To summarize, [Zn(CH_3_COO)(cur)(bpy)](**1**)·CH_3_OH·2H_2_O and [Zn(PhCOO)(cur)(bpy)] (**2**)·CH_3_OH were crystallized from methanol solutions. Good quality single crystals were prepared and subjected to X-ray structural analysis. The complexes **1** and **2** are the second only examples of X-ray structurally characterized zinc(II) curcuminate compounds. The five-fold coordination environments of zinc(II) in the pair, although similar, exhibit minor differences. The spatial distribution of the monodentate carboxylate and bidentate chelating 2,2′-bipyridine and curcuminate is such that the O_3_N_2_ donor set in the case of acetate complex **1** defines a distorted square pyramid, whereas in complex with benzoate **2,** a trigonal bipyramid is defined. **1**·CH_3_OH·2H_2_O and **2**·CH_3_OH display different packing arrangements in the solid state. Hydrogen-bonding interactions govern the packing. The acetate-curcuminate Zn(II) compound does not have the anticipated water solubility. Both compounds are poorly soluble in aprotic solvents. Surprisingly, unlike the related heteroleptic Zn(II) complex with curcuminate, which displays stability under physiological conditions, the coordination of curcuminate to Zn(II) in **1** and **2** does not prevent its degradation in alkaline media. Both complexes are fluorophores with a green emission after excitation at 430 nm. The synthesis of several similar curcumin metal complexes with other carboxylate ligands is underway, and investigation of their biological activity is under consideration.

## Figures and Tables

**Table 1:** Relevant structural parameters (Å, °) for [Zn(CH_3_COO)(cur)(bpy)] (**1**)·CH_3_OH·2H_2_O and [Zn(PhCOO)(cur)(bpy)](**2**)·CH_3_OH.

1		2	
Zn(1)–O(7)	1.973(1)	Zn(1)–O(7)	1.987(3)
Zn(1)–O(1)	2.016(1)	Zn(1)–O(1)	2.007(3)
Zn(1)–O(2)	2.001(1)	Zn(1)–O(2)	2.051(3)
Zn(1)–N(1)	2.162(2)	Zn(1)–N(1)	2.121(4)
Zn(1)–N(2)	2.123(1)	Zn(1)–N(2)	2.112(4)
Zn(1)–O(8)	3.303(2)	Zn(1)–O(8)	3.127(3)
C(8)–O(7)	1.263(2)	C(31)–O(7)	1.266(5)
C(8)–O(8)	1.243(2)	C(31)–O(8)	1.242(5)
O(7)–Zn(1)–O(1)	100.79(6)	N(1)–Zn(1)–O(2)	162.9(1)
O(7)–Zn(1)–O(2)	101.48(6)	N(1)–Zn(1)–N(2)	77.0(2)
O(7)–Zn(1)–N(1)	99.73(6)	N(1)–Zn(1)–O(1)	90.5(1)
O(7)–Zn(1)–N(2)	123.32(6)	N(1)–Zn(1)–O(7)	98.6(1)
O(1)–Zn(1)–N(1)	158.86(6)	O(1)–Zn(1)–O(7)	127.3(1)
O(2)–Zn(1)–N(2)	134.18(6)	O(7)–Zn(1)–N(2)	94.8(1)
O(1)–Zn(1)–O(2)	92.22(5)	N(2)–Zn(1)–O(1)	124.8(1)
O(2)–Zn(1)–N(1)	88.61(5)	O(2)–Zn(1)–O(1)	89.75(1)
N(1)–Zn(1)–N(2)	75.82(6)	O(2)–Zn(1)–O(7)	94.8(1)
N(2)–Zn(1)–O(1)	88.66(5)	O(2)–Zn(1)–N(2)	88.9(1)

**Table 2:** Hydrogen-bonding interactions in [Zn(PhCOO)(cur)(bpy)](**2**)·CH_3_OH ^(a)^.

Groups Engaged in Interaction ^(b)^	Donor Atom Acceptor ^(c)^	Length (Å)
OH·CH_3_OH	O(6)···O(9) ^i^	2.715(5)
OH···O(cur^−^)	O(4)···O(1) ^ii^	2.788(4)
CH_3_OH···COO^−^	O(9)···O(8)	2.709(5)

^(a)^ The distances may be compared to the sum of the van der Waals radii for oxygen atoms, 3.04 Å [53].^(b)^ OH is a hydroxyl group of the cur^−^ ligand, whereas O(cur^−^) denotes one of its two coordinating oxygens. ^(c)^ Symmetry codes are: (i) *x* + 1, *y*, and *z*; (ii) *x* − 0.5, −*y* + 0.5, and *z* − 0.5.

**Table 3:** Hydrogen-bonding interactions in [Zn(CH_3_COO)(cur)(bpy)](**1**)·CH_3_OH·2H_2_O ^(a)^.

Groups Engaged in Interaction ^(b)^	Donor Atom Acceptor ^(c)^	Length (Å)
OH···COO^−^	O(6)···O(8) ^i^	2.653(2)
OH···H_2_O	O(4)···O(12) ^ii^	2.729(2)
OH···H_2_O	O(6)···O(12) ^iii^	3.012(2)
CH_3_OH···COO^−^	O(10)···O(8) ^i^	2.787(3)
CH_3_OH···H_2_O	O(10)···O(11) ^iv^	2.702(3)
H_2_O···OCH_3_	O(12)···O(3) ^ii^	3.026(2)

^(a)^ Distances may be compared to the sum of the van der Waals radii for oxygen atoms, 3.04 Å [53]. ^(b)^ OH and OCH_3_ stand for hydroxyl and methoxy functional groups of the cur^−^ ligand, respectively. ^(c)^ Symmetry codes are: (i) *−x* + 1.5, *y* + 0.5, −*z* − 1; (ii) *−x* + 0.5, *y* − 0.5, −*z*; (iii) *−x* + 1.5, *y* + 0.5, −*z* − 1; (iv) *−x* + 0.5, *y* + 0.5, −*z* − 1.

## Supplementary Materials

The following are available online at https://www.mdpi.com/1420-3049/24/14/2540/s1. Figure S1. Time-dependent UV-Vis spectra of curH in DMSO. Figure S2. Time-dependent UV-Vis spectra of [Zn(CH_3_COO)(cur)(bpy)](**1**) in DMSO. Figure S3. Time-dependent UV-Vis spectra of [Zn(PhCOO)(cur)(bpy)](**2**) in DMSO. Figure S4. Time-dependent UV-Vis spectra of curH in 90 vol.% 100 mM NaCl - 10 vol. % DMSO. Figure S5. Time-dependent UV-Vis spectra of [Zn(CH_3_COO)(cur)(bpy)](**1**) in 90 vol.% 100 mM NaCl - 10 vol. % DMSO. Figure S6. Time-dependent UV-Vis spectra of [Zn(PhCOO)(cur)(bpy)](**2**) in 90 vol.% 100 mM NaCl - 10 vol. % DMSO. Figure S7. Time-dependent UV-Vis spectra of curH in 90 vol.% PBS (pH = 7.0) - 10 vol.% DMSO solution. Figure S8. Time-dependent UV-Vis spectra of [Zn(CH_3_COO)(cur)(bpy)](**1**) in 90 vol.% PBS (pH = 7.0) - 10 vol.% DMSO solution. Figure S9. Time-dependent UV-Vis spectra of [Zn(PhCOO)(cur)(bpy)](**2**) in 90 vol.% PBS (pH = 7.0) - 10 vol.% DMSO solution. Figure S10. 1H NMR spectrum (500 MHz) of [Zn(CH_3_COO)(cur)(bpy)](**1**) in DMSO-d6. Figure S11. 1H NMR spectrum (500 MHZ) of [Zn(PhCOO)(cur)(bpy)](**2**) in DMSO-d6. Figure S12. 1H NMR spectrum (500 MHz) of [Zn(PhCOO)_2_] in DMSO-d6. Figure S13. 2D [^1^H,^1^H] COSY NMR (500 MHZ) spectrum of [Zn(CH_3_COO)(cur)(bpy)](**1**) in DMSO-d6. Figure S14. 2D [^1^H,^1^H] COSY NMR (500 MHZ) spectrum of [Zn(PhCOO)(cur)(bpy)](**2**) in DMSO-d6. Figure S15. ATR-IR spectrum of curH. Figure S16. ATR-IR spectrum of [Zn(CH_3_COO)(cur)(bpy)](**1**)·CH_3_OH·2H_2_O. Figure S17. ATR-IR spectrum of [Zn(PhCOO)(cur)(bpy)](**2**)·CH_3_OH. Figure S18. ATR-IR spectrum of [Zn(PhCOO)_2_]. Figure S19. TGA curve of [Zn(CH_3_COO)(cur)(bpy)](**1**)·CH_3_OH·2H2O, recorded after being exposed to air. Figure S20. TGA curve of crystals of [Zn(PhCOO)(cur)(bpy)](**2**)·CH_3_OH, recorded after being exposed to air. 23 Figure S21. TGA curve of [Zn(PhCOO)_2_].
