# Supplementary material for: Zn(II) Curcuminate Complexes with 2,2′-bipyridine and Carboxylates
Source: Molecules. 2019 Jul 11;24(14):2540. doi: 10.3390/molecules24142540 (PMC6680645; doi:10.3390/molecules24142540)
Supplement: Supplementary file 1 [file molecules-24-02540-s001.pdf]

Electronic Supplementary Material

Zn(II) curcuminate complexes with 2,2′-bipyridine and carboxylates

Sabina Graber[1*], Barbara Modec [1*]

[1] University of Ljubljana, Faculty of Chemistry and Chemical Technology, Večna pot 113, 1000 Ljubljana, Slovenia sabina.grabner@fkkt.uni-lj.si
Correspondence: barbara.modec@fkkt.uni-lj.si; sabina.grabner@fkkt.uni-lj.si

Index

p. 3 **Figure S1**. Time-dependent UV-Vis spectra of curH in DMSO.
p. 4 **Figure S2**. Time-dependent UV-Vis spectra of [Zn(CH_3COO)(cur)(bpy)](**1**) in DMSO.
p. 5 **Figure S3**. Time-dependent UV-Vis spectra of [Zn(PhCOO)(cur)(bpy)](**2**) in DMSO.
p. 6 **Figure S4**. Time-dependent UV-Vis spectra of curH in 90 vol.% 100 mM NaCl - 10 vol. % DMSO.
p. 7 **Figure S5**. Time-dependent UV-Vis spectra of [Zn(CH_3COO)(cur)(bpy)](**1**) in 90 vol.% 100 mM NaCl - 10 vol. % DMSO.
p. 8 **Figure S6**. Time-dependent UV-Vis spectra of [Zn(PhCOO)(cur)(bpy)](**2**) in 90 vol.% 100 mM NaCl - 10 vol. % DMSO.
p. 9 **Figure S7**. Time-dependent UV-Vis spectra of curH in 90 vol.% PBS (pH = 7.0) - 10 vol.% DMSO solution.
p. 10 **Figure S8**. Time-dependent UV-Vis spectra of [Zn(CH_3COO)(cur)(bpy)](**1**) in 90 vol.% PBS (pH = 7.0) - 10 vol.% DMSO solution.
p. 11 **Figure S9**. Time-dependent UV-Vis spectra of [Zn(PhCOO)(cur)(bpy)](**2**) in 90 vol.% PBS (pH = 7.0) - 10 vol.% DMSO solution.
p. 12 **Figure S10**. ^{1}H NMR spectrum (500 MHz) of [Zn(CH_3COO)(cur)(bpy)](**1**) in DMSO-d_6.
p. 13 **Figure S11**. ^{1}H NMR spectrum (500 MHZ) of [Zn(PhCOO)(cur)(bpy)](**2**) in DMSO-d_6.
p. 14 **Figure S12**. ^{1}H NMR spectrum (500 MHz) of [Zn($PhCOO)_2$] in DMSO-d_6.
p. 15 **Figure S13**. 2D [^{1}H,^{1}H] COSY NMR (500 MHZ) spectrum of [Zn(CH_3COO)(cur)(bpy)](**1**) in DMSO-d_6.
p. 16 **Figure S14**. 2D [^{1}H,^{1}H] COSY NMR (500 MHZ) spectrum of [Zn(PhCOO)(cur)(bpy)](**2**) in DMSO-d_6.
p. 17 **Figure S15**. ATR-IR spectrum of curH.
p. 18 **Figure S16**. ATR-IR spectrum of [Zn(CH_3COO)(cur)(bpy)](**1**)·CH_3OH·$2H_2O$.
p. 19 **Figure S17**. ATR-IR spectrum of [Zn(PhCOO)(cur)(bpy)](**2**)·CH_3OH.
p. 20 **Figure S18**. ATR-IR spectrum of [Zn($PhCOO)_2$].
p. 21 **Figure S19**. TGA curve of [Zn(CH_3COO)(cur)(bpy)](**1**)·CH_3OH·$2H_2O$, recorded after being exposed to air.
p. 22 **Figure S20**. TGA curve of crystals of [Zn(PhCOO)(cur)(bpy)](**2**)·CH_3OH, recorded after being exposed to air.
p. 23 **Figure S21**. TGA curve of [Zn($PhCOO)_2$].

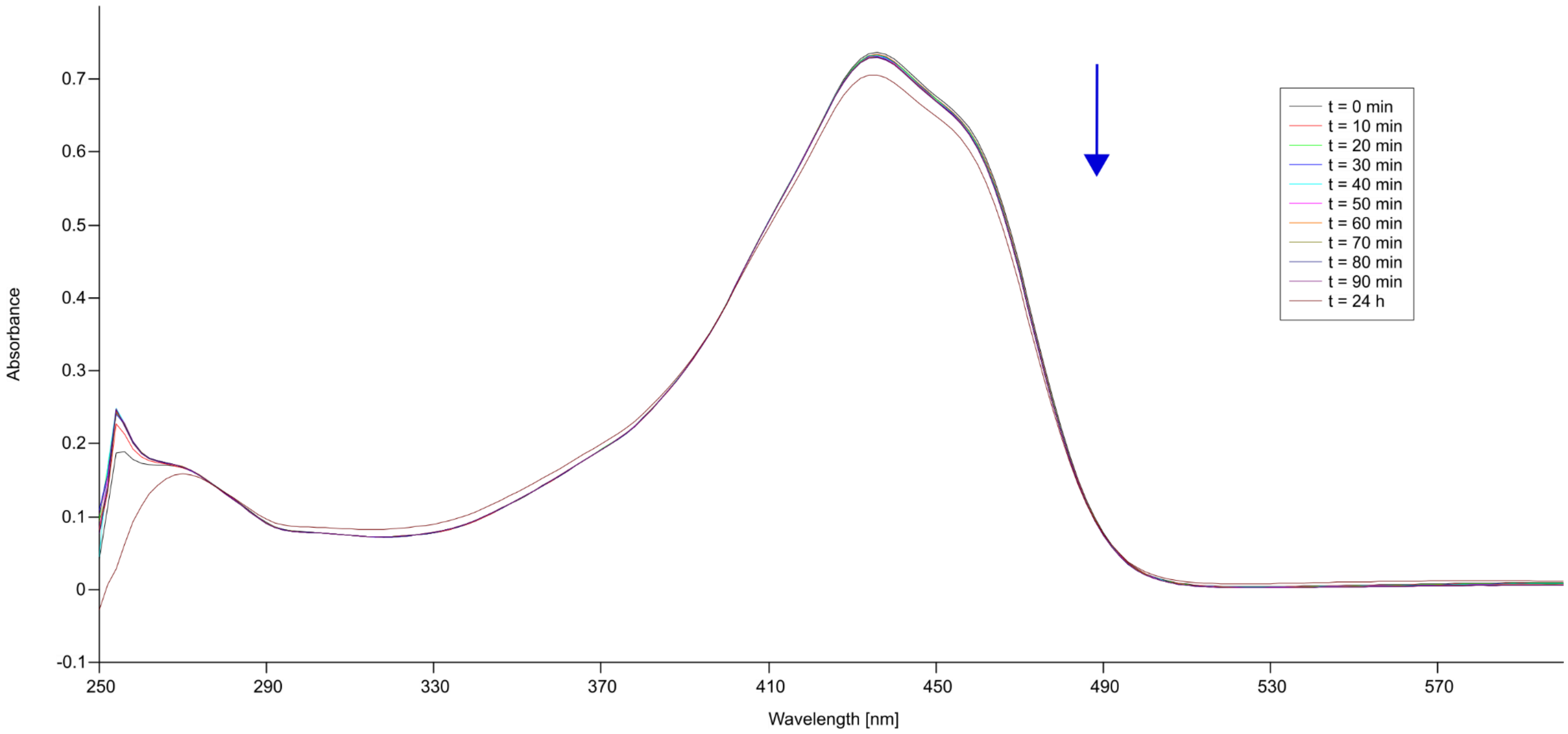

Figure S1. Time-dependent UV-Vis spectra of curH in DMSO.

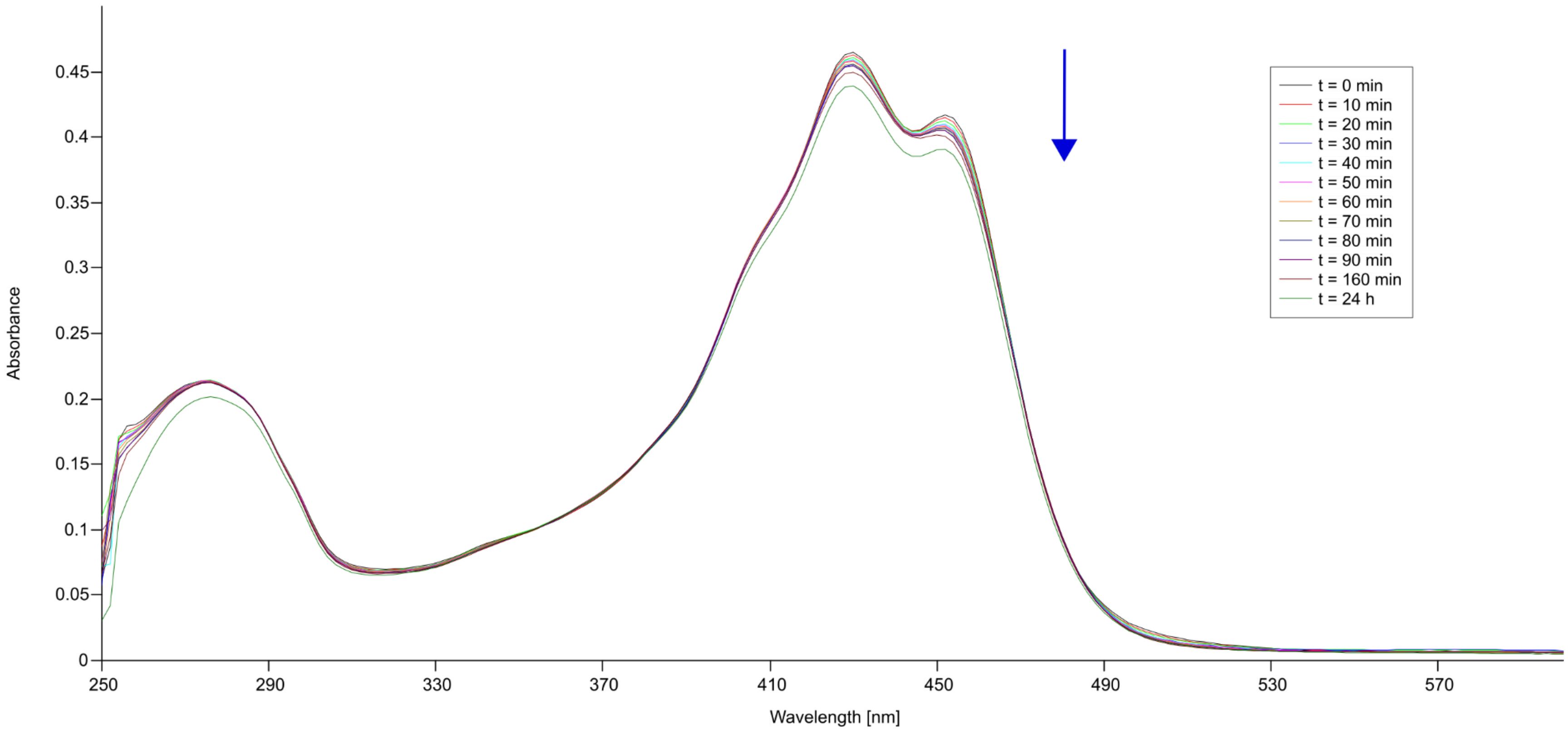

Figure S2. Time-dependent UV-Vis spectra of [Zn(CH_3COO)(cur)(bpy)](**1**) in DMSO.

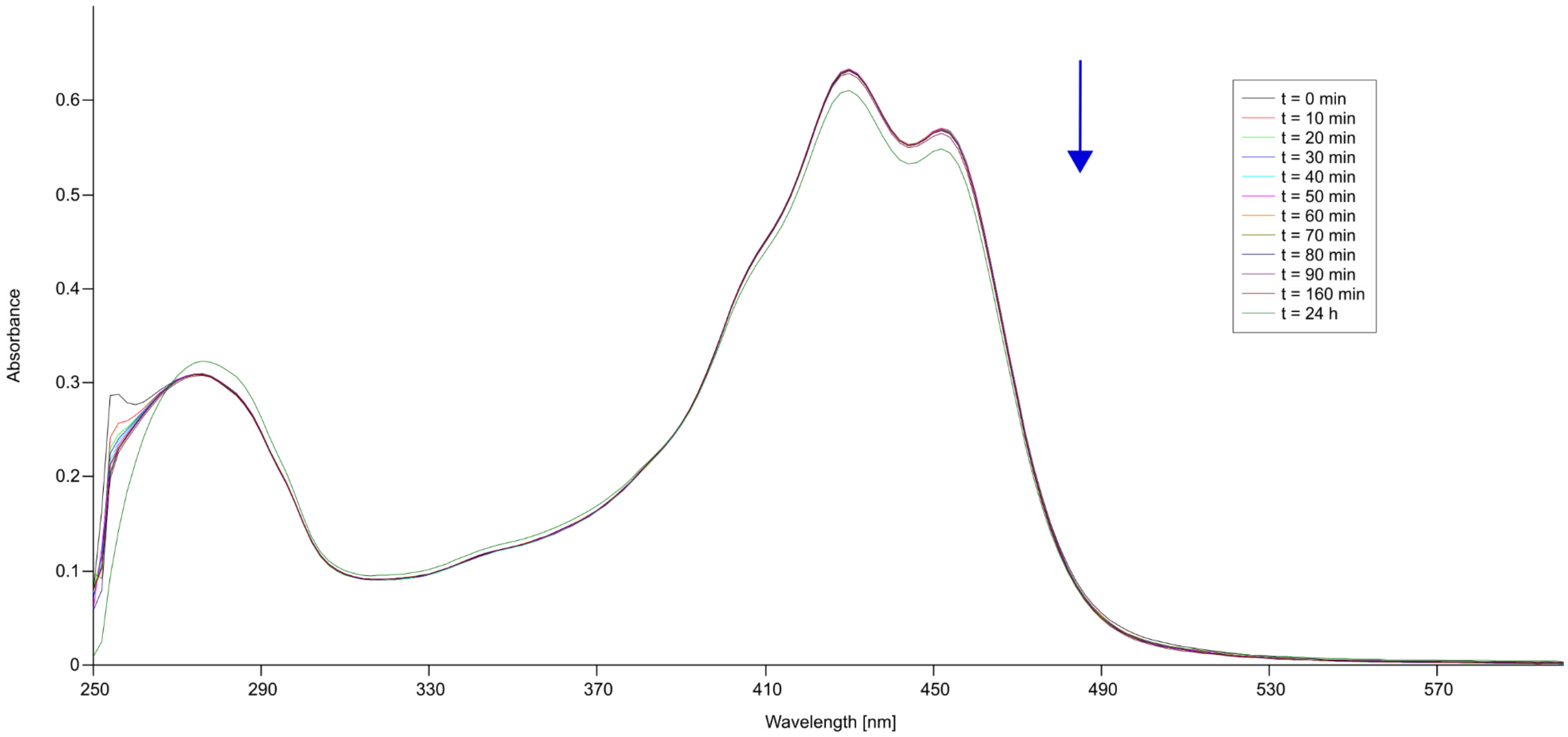

Figure S3. Time-dependent UV-Vis spectra of [Zn(PhCOO)(cur)(bpy)](**2**) in DMSO.

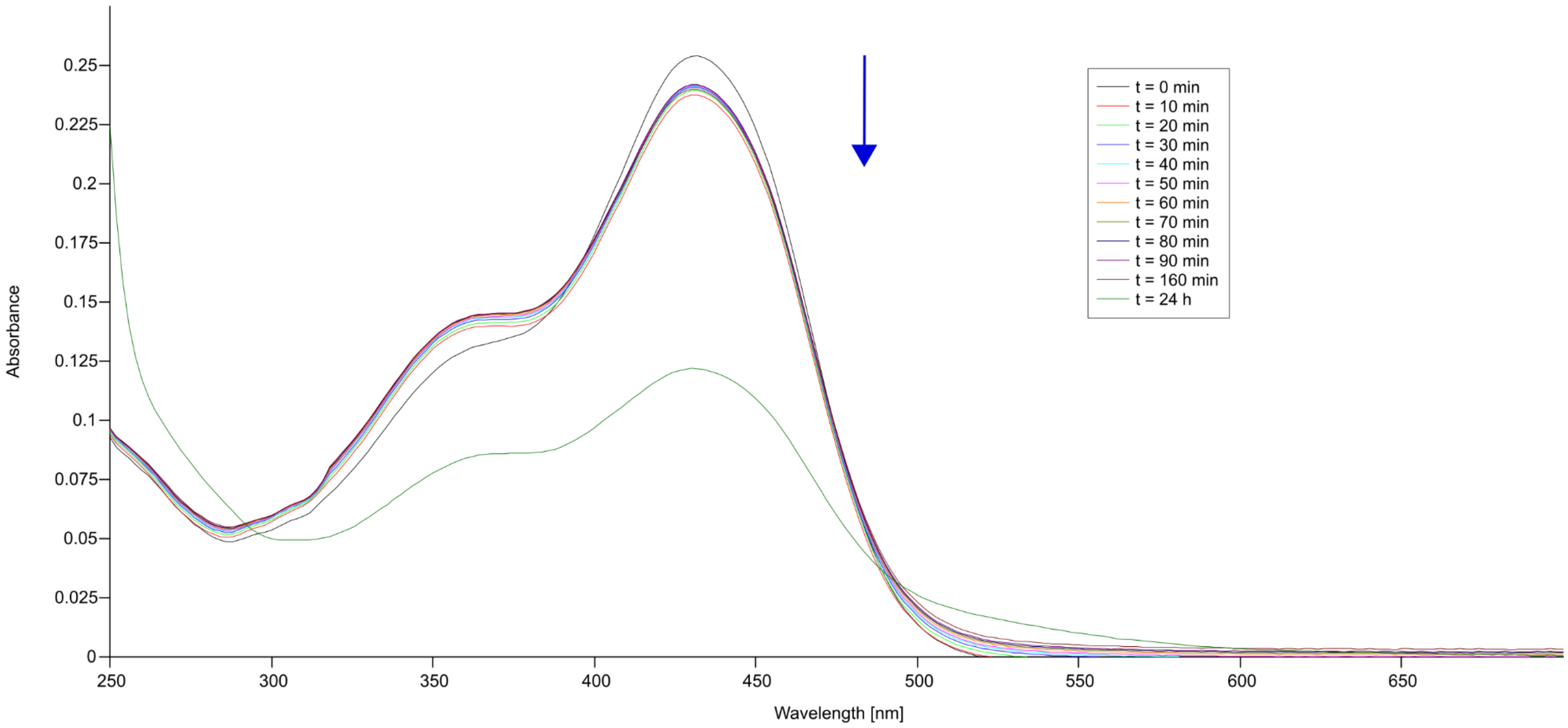

Figure S4. Time-dependent UV-Vis spectra of curH in 90 vol.% 100 mM NaCl - 10 vol. % DMSO.

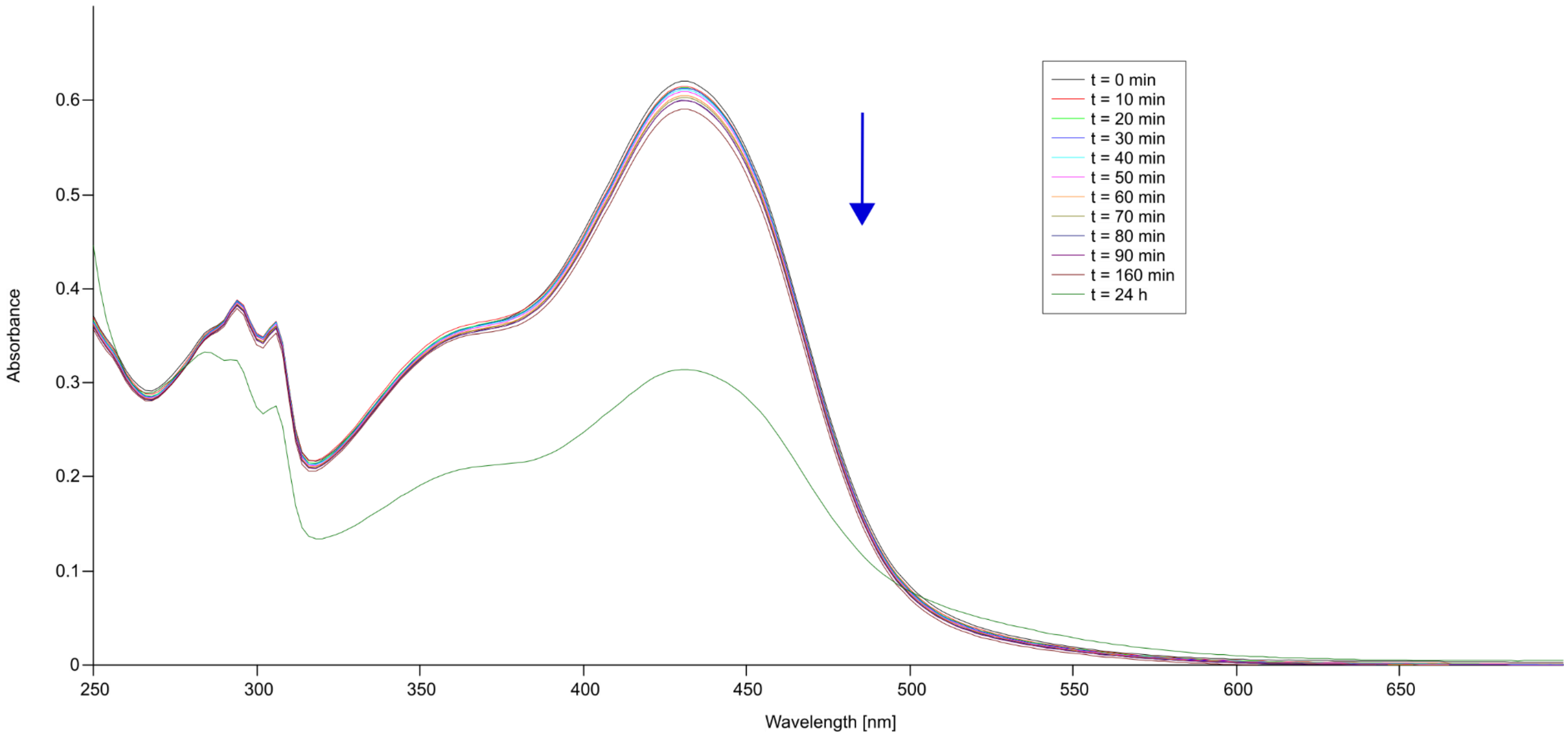

Figure S5. Time-dependent UV-Vis spectra of [Zn(CH_3COO)(cur)(bpy)](**1**) in 90 vol.% 100 mM NaCl - 10 vol. % DMSO.

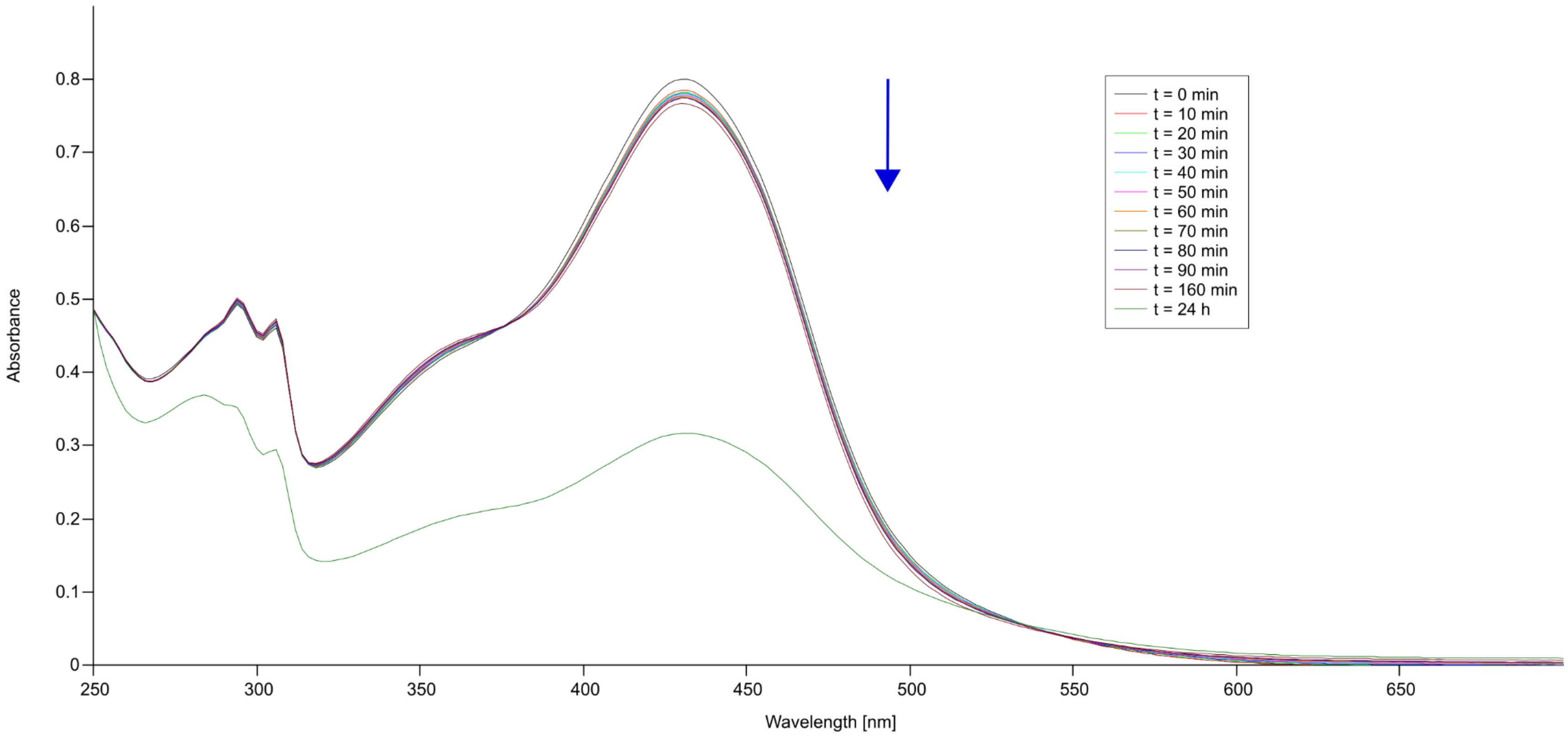

Figure S6. Time-dependent UV-Vis spectra of [Zn(PhCOO)(cur)(bpy)](**2**) in 90 vol.% 100 mM NaCl - 10 vol. % DMSO.

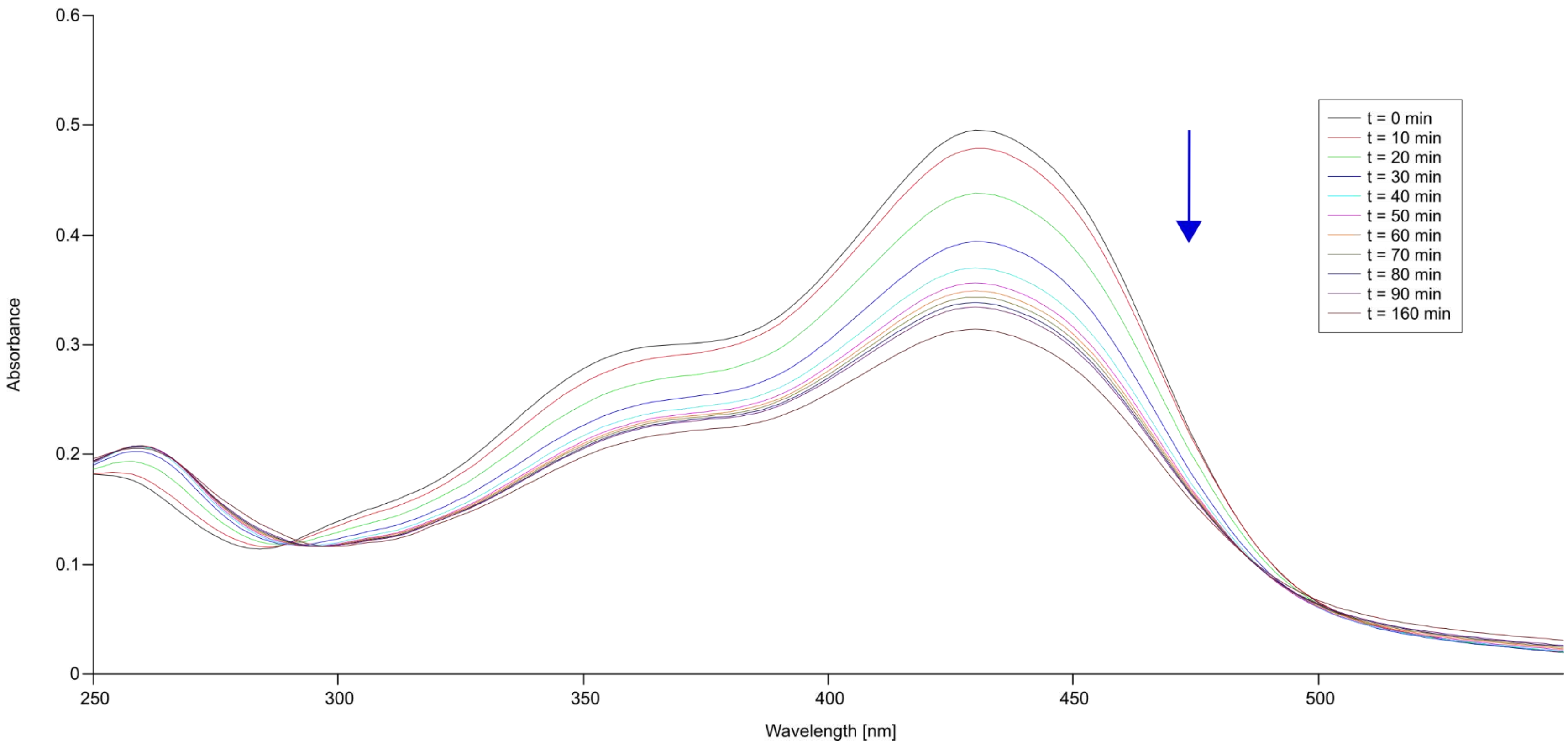

Figure S7. Time-dependent UV-Vis spectra of curH in 90 vol.% PBS (pH = 7.0) - 10 vol.% DMSO solution.

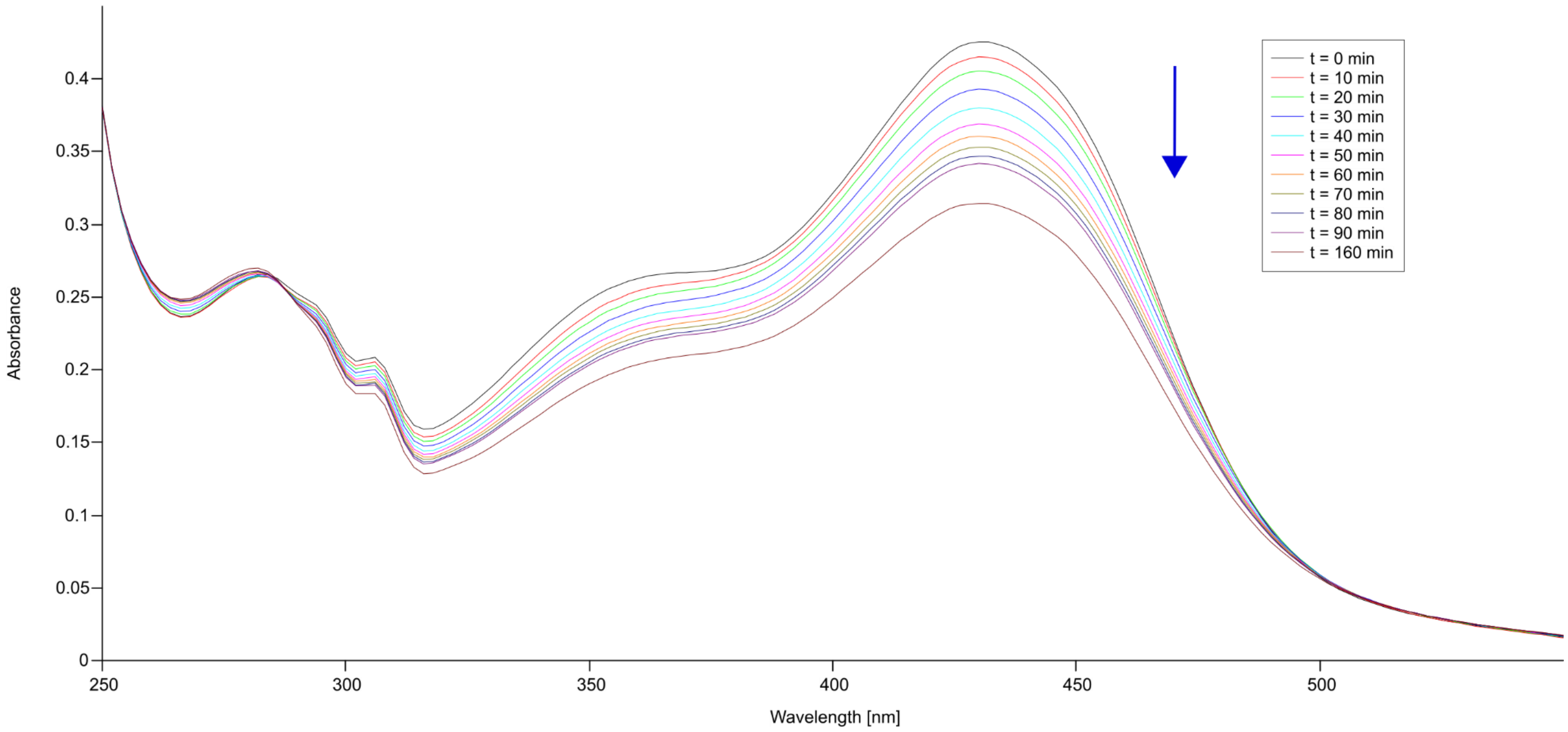

Figure S8. Time-dependent UV-Vis spectra of [Zn(CH_3COO)(cur)(bpy)](**1**) in 90 vol.% PBS (pH = 7.0) - 10 vol.% DMSO solution.

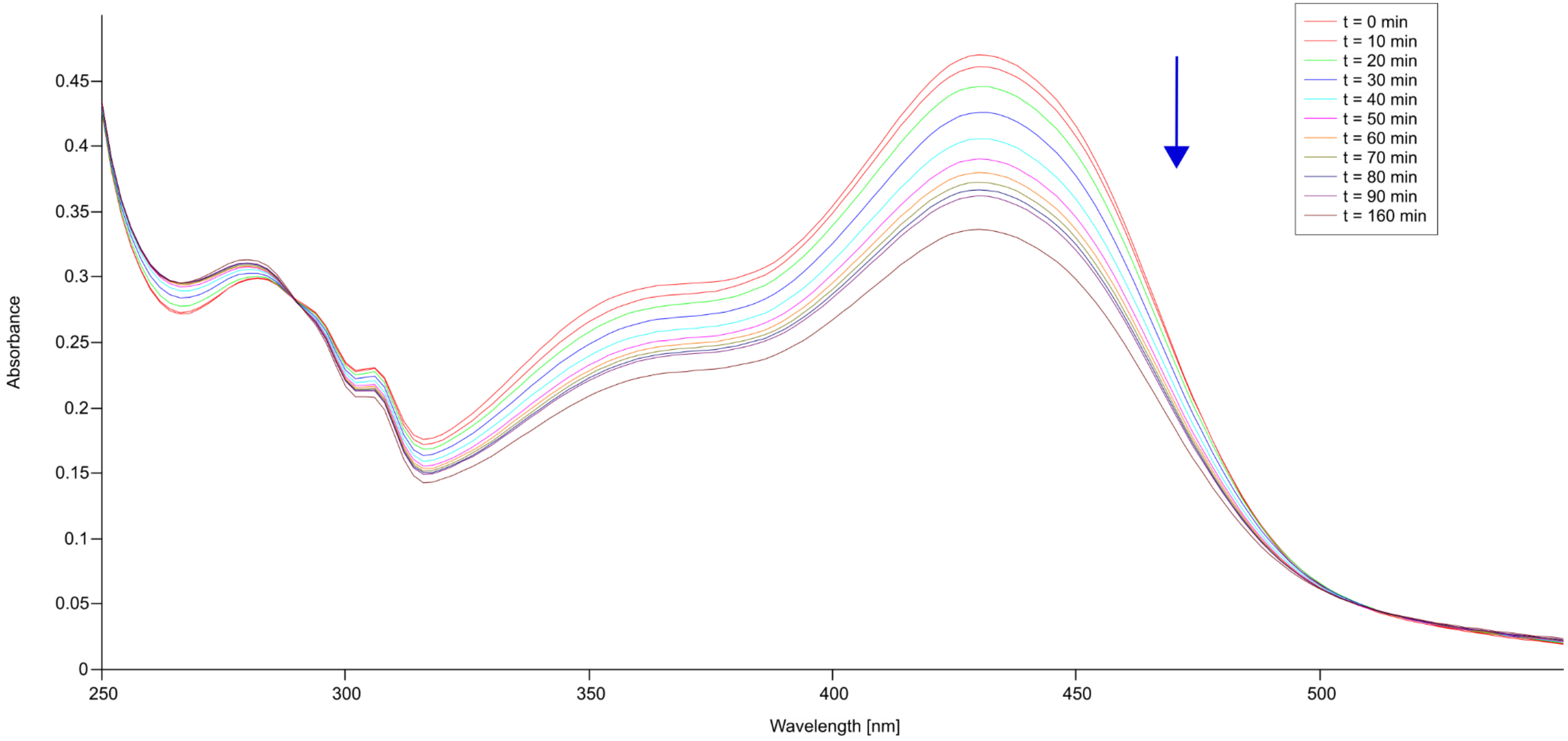

Figure S9. Time-dependent UV-Vis spectra of [Zn(PhCOO)(cur)(bpy)](**2**) in 90 vol.% PBS (pH = 7.0) - 10 vol.% DMSO solution.

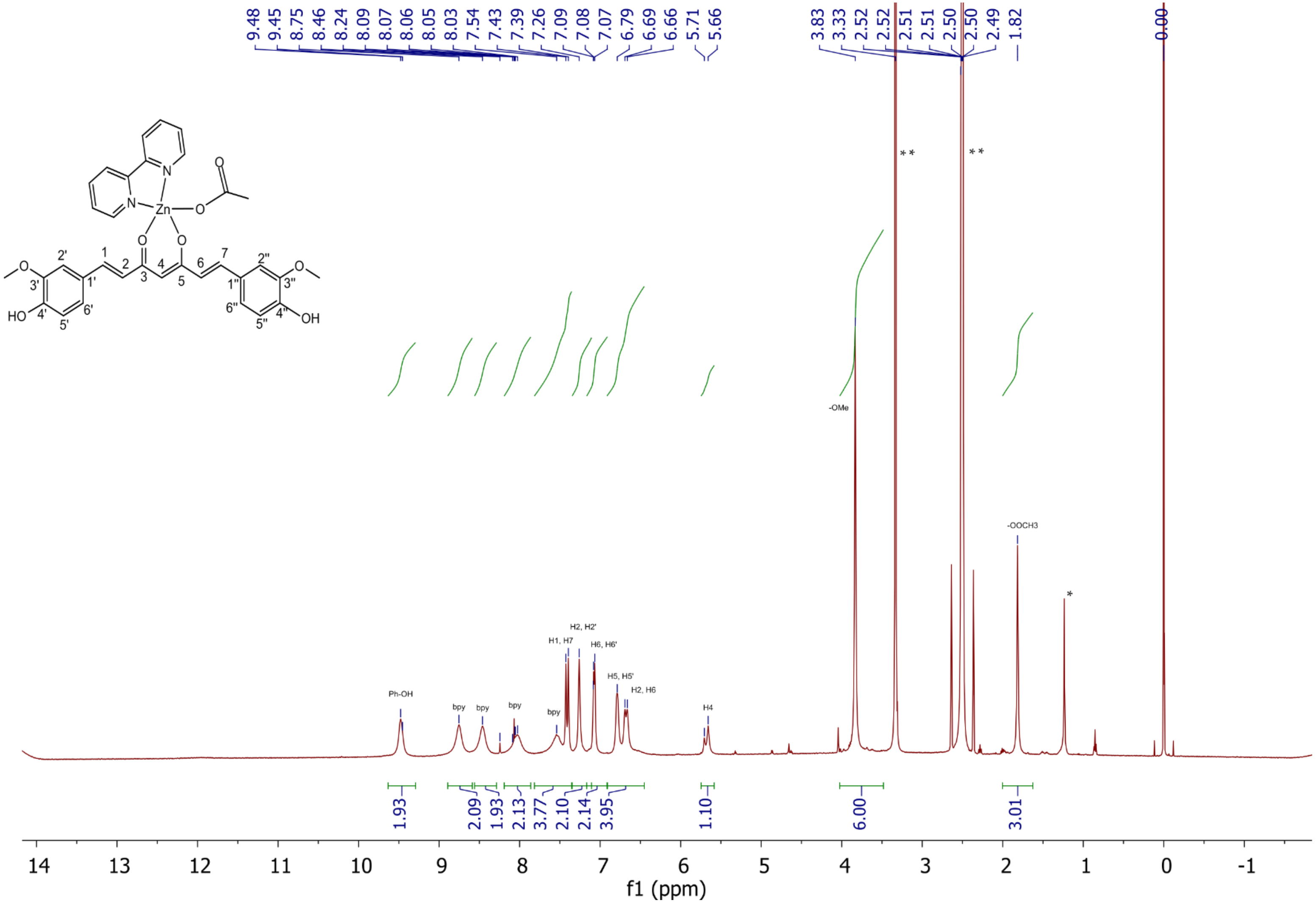

Figure S10. ^{1}H NMR spectrum (500 MHz) of [Zn(CH_3COO)(cur)(bpy)](**1**) in DMSO-d_6. The solvent and water peaks are marked with asterisks **, impurity from a vial with *.

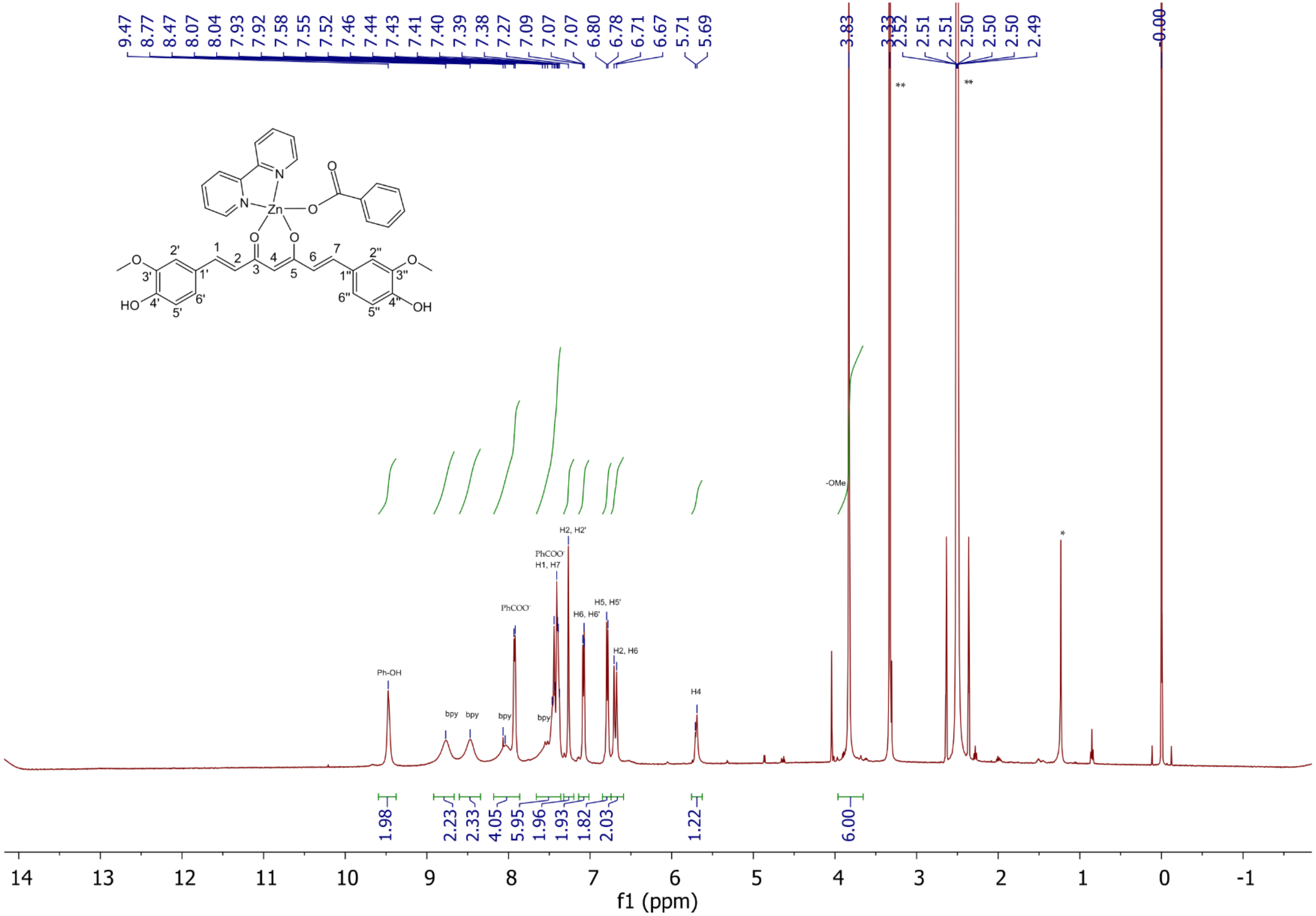

Figure S11. ^{1}H NMR spectrum (500 MHZ) of [Zn(PhCOO)(cur)(bpy)](**2**) in DMSO-d_6. The solvent and water peaks are marked with asterisks **, impurity from a vial with *.

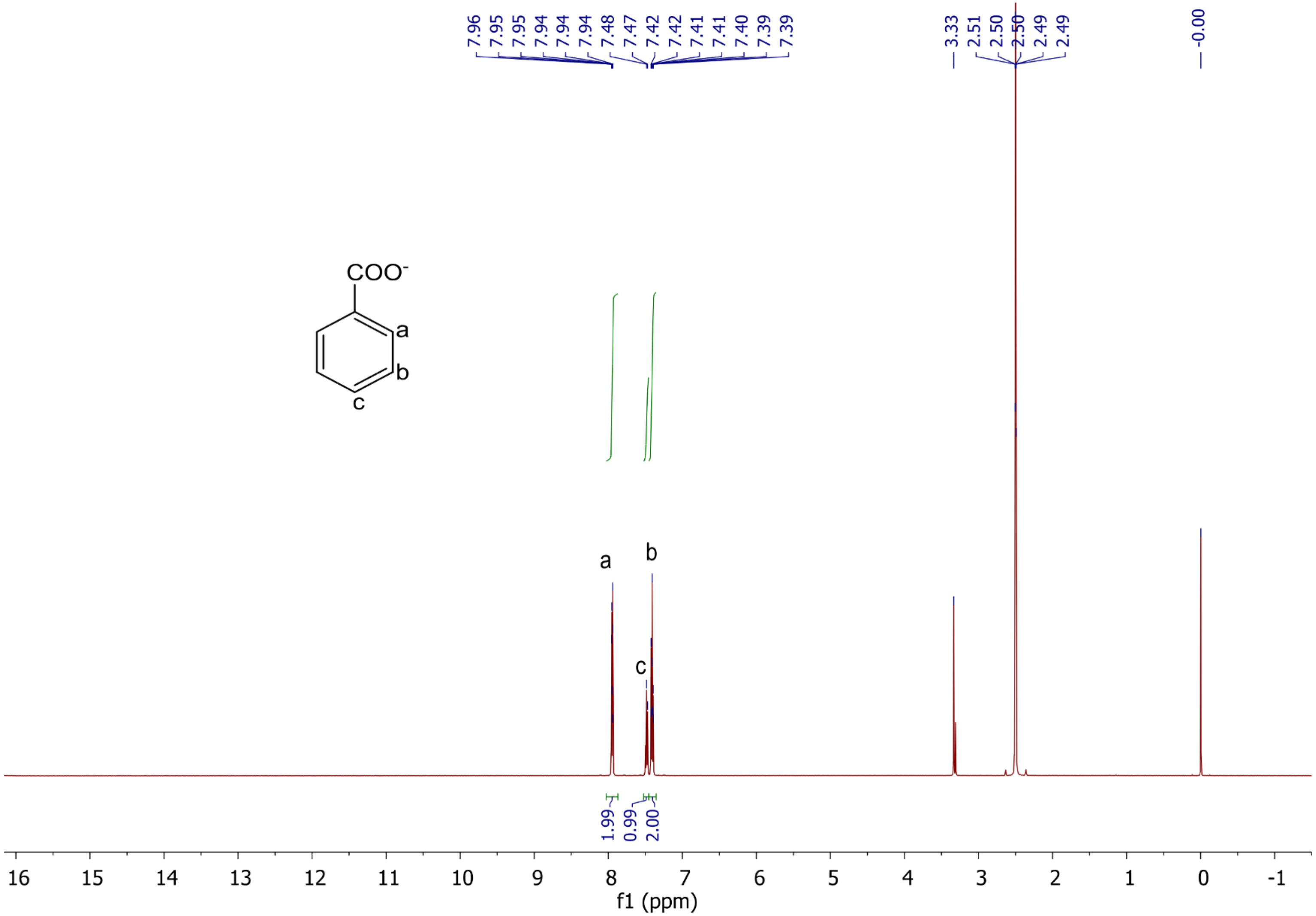

Figure S12. ^{1}H NMR spectrum (500 MHz) of [Zn(PhCOO)$_2$] in DMSO-d_6.

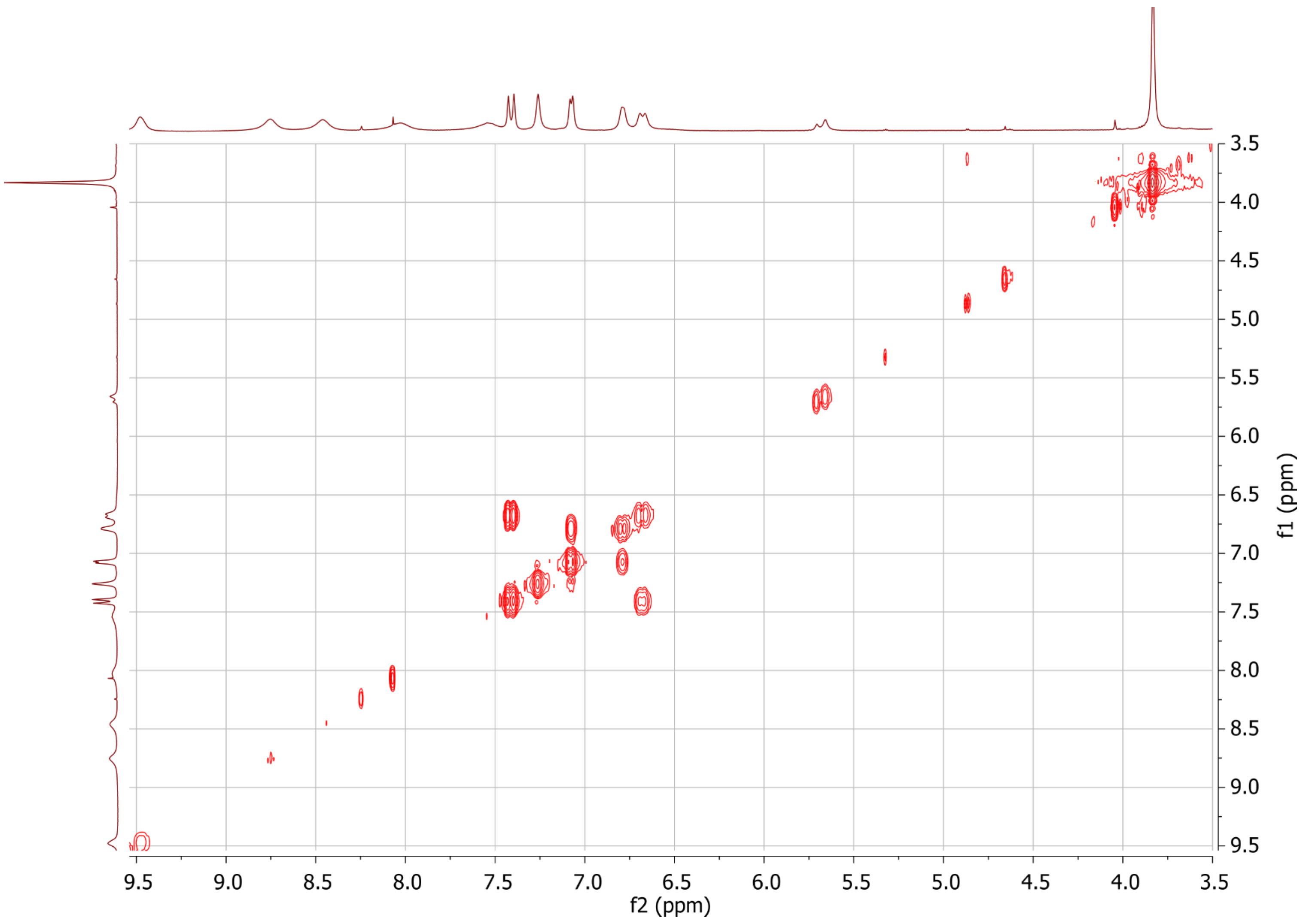

Figure S13. 2D [^{1}H,^{1}H] COSY NMR (500 MHZ) spectrum of [Zn(CH_3COO)(cur)(bpy)](**1**) in DMSO-d_6.

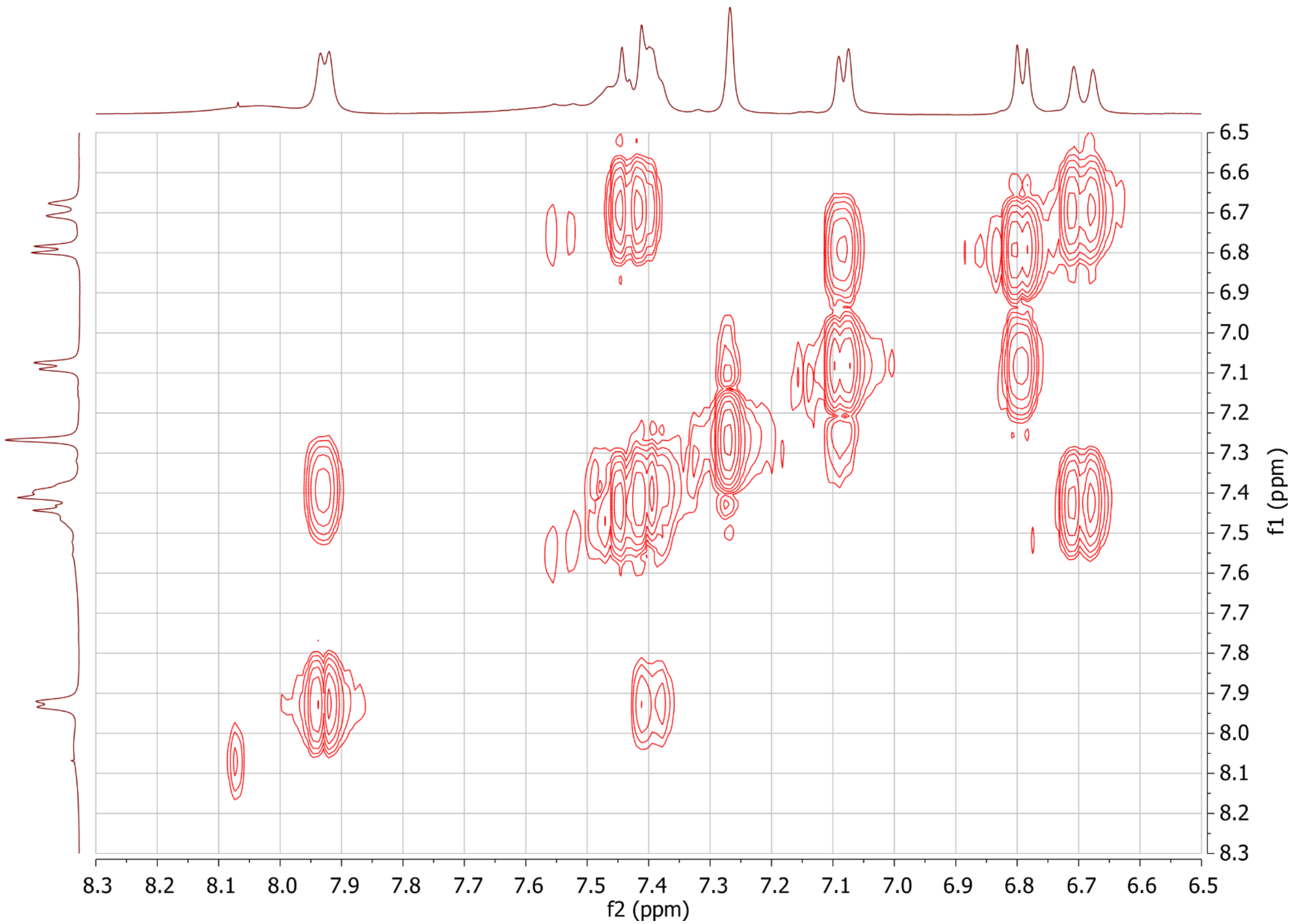

Figure S14. 2D [^{1}H,^{1}H] COSY NMR (500 MHZ) spectrum of [Zn(PhCOO)(cur)(bpy)](**2**) in DMSO-*d*$_6$.

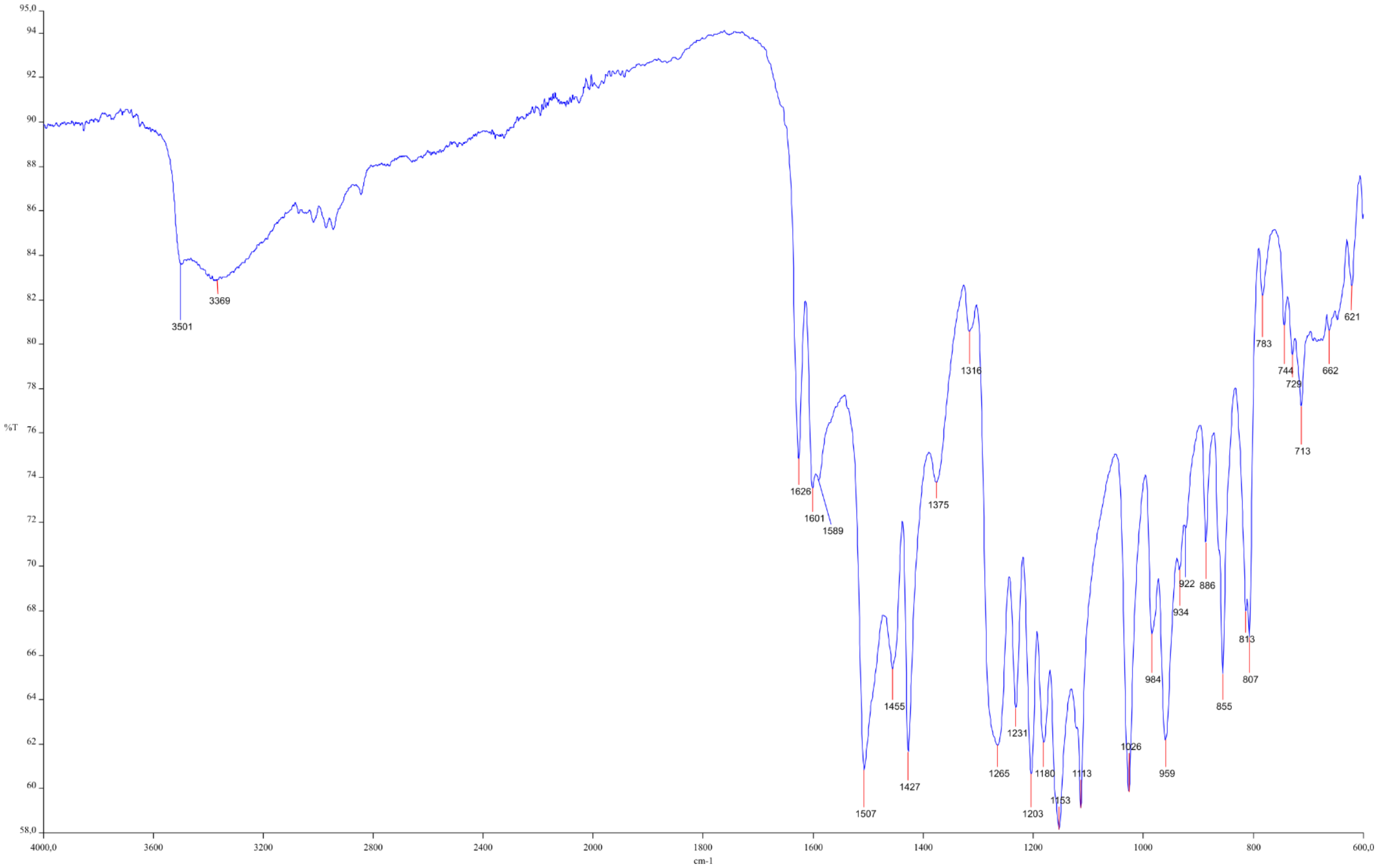

Figure S15. ATR-IR spectrum of curH.

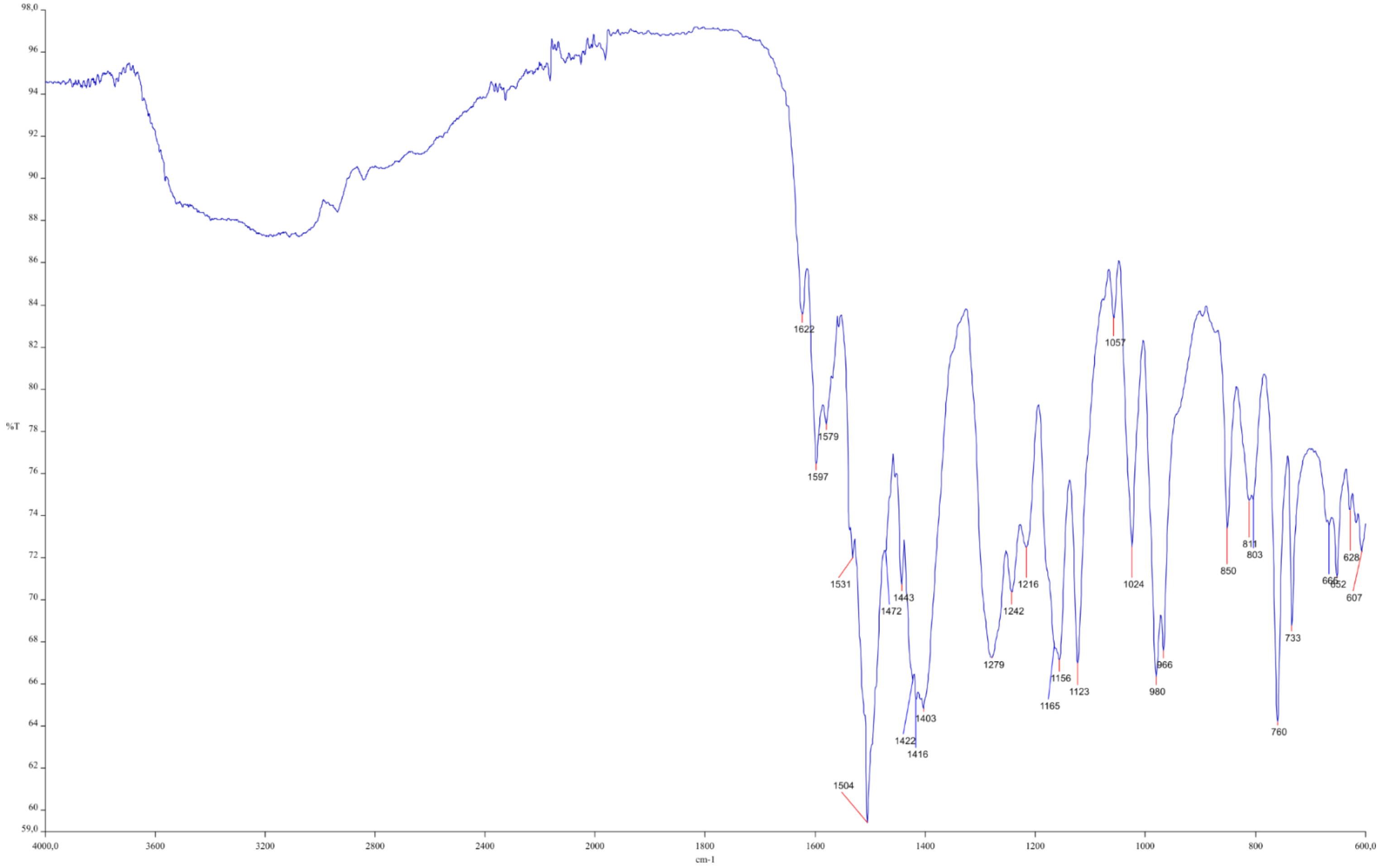

Figure S16. ATR-IR spectrum of [Zn(CH_3COO)(cur)(bpy)](**1**)·CH_3OH·$2H_2O$.

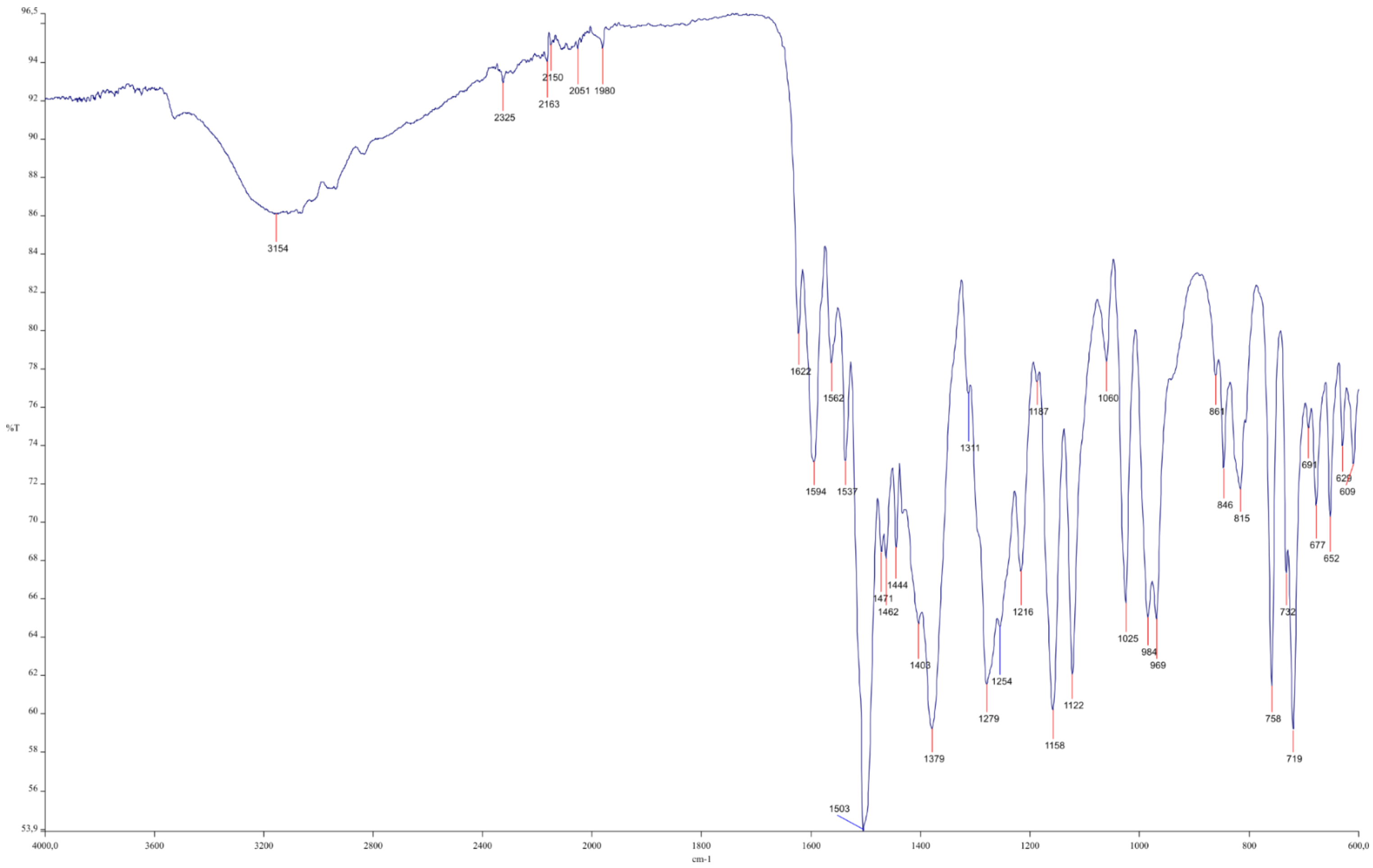

Figure S17. ATR-IR spectrum of [Zn(PhCOO)(cur)(bpy)](**2**)·CH_3OH.

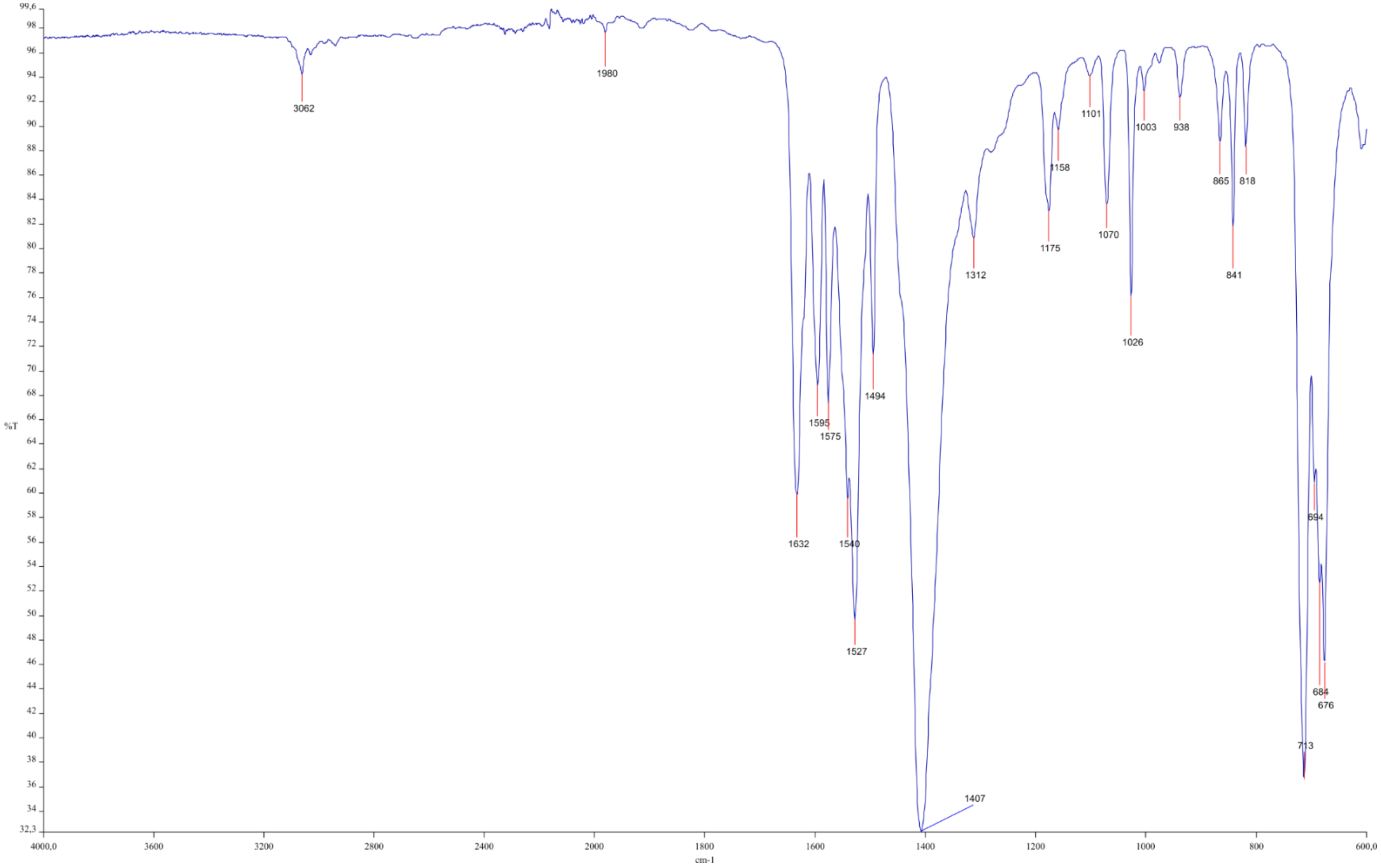

Figure S18. ATR-IR spectrum of [Zn(PhCOO)$_2$].

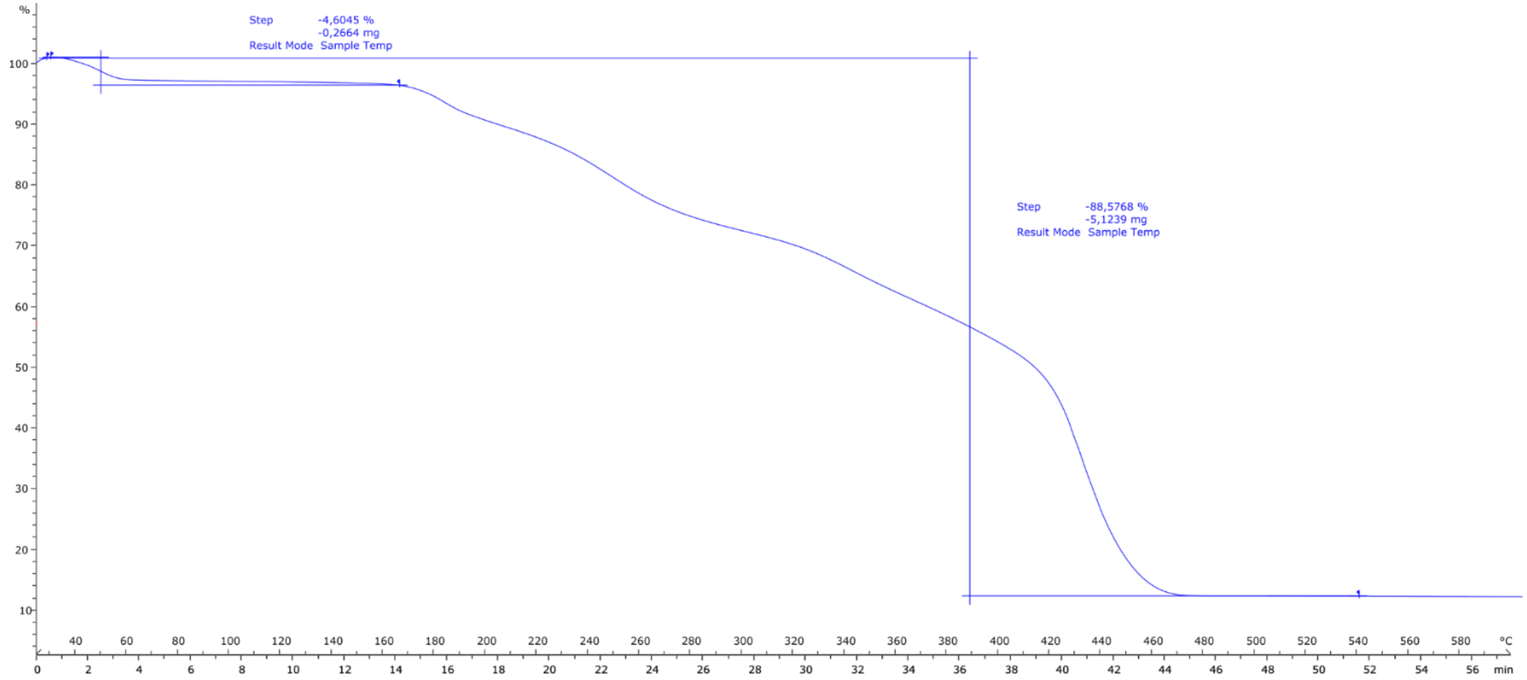

Figure S19. TGA curve of crystals of [Zn(CH_3COO)(cur)(bpy)](**1**)·CH_3OH·2H_2O, recorded after being exposed to air.

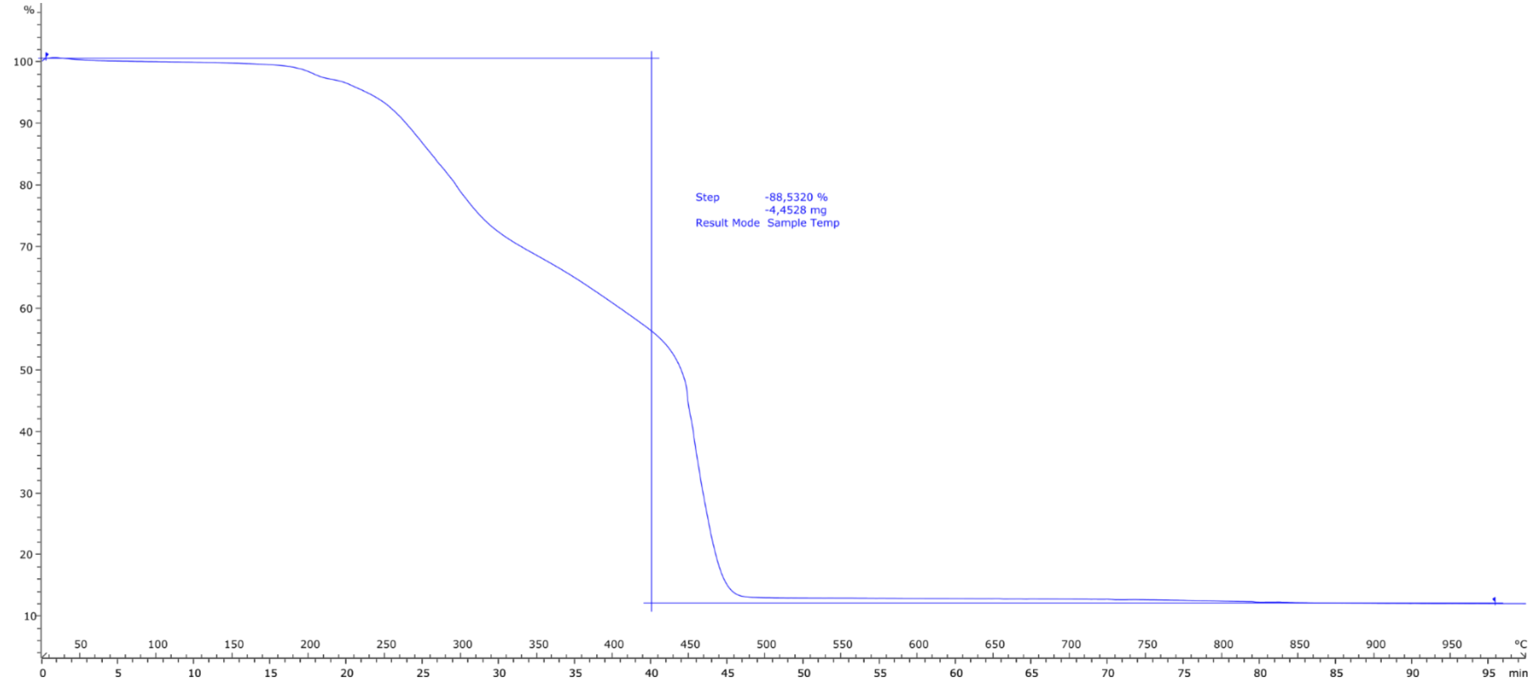

Figure S20. TGA curve of crystals of [Zn(PhCOO)(cur)(bpy)](**2**)·CH_3OH, recorded after being exposed to air.

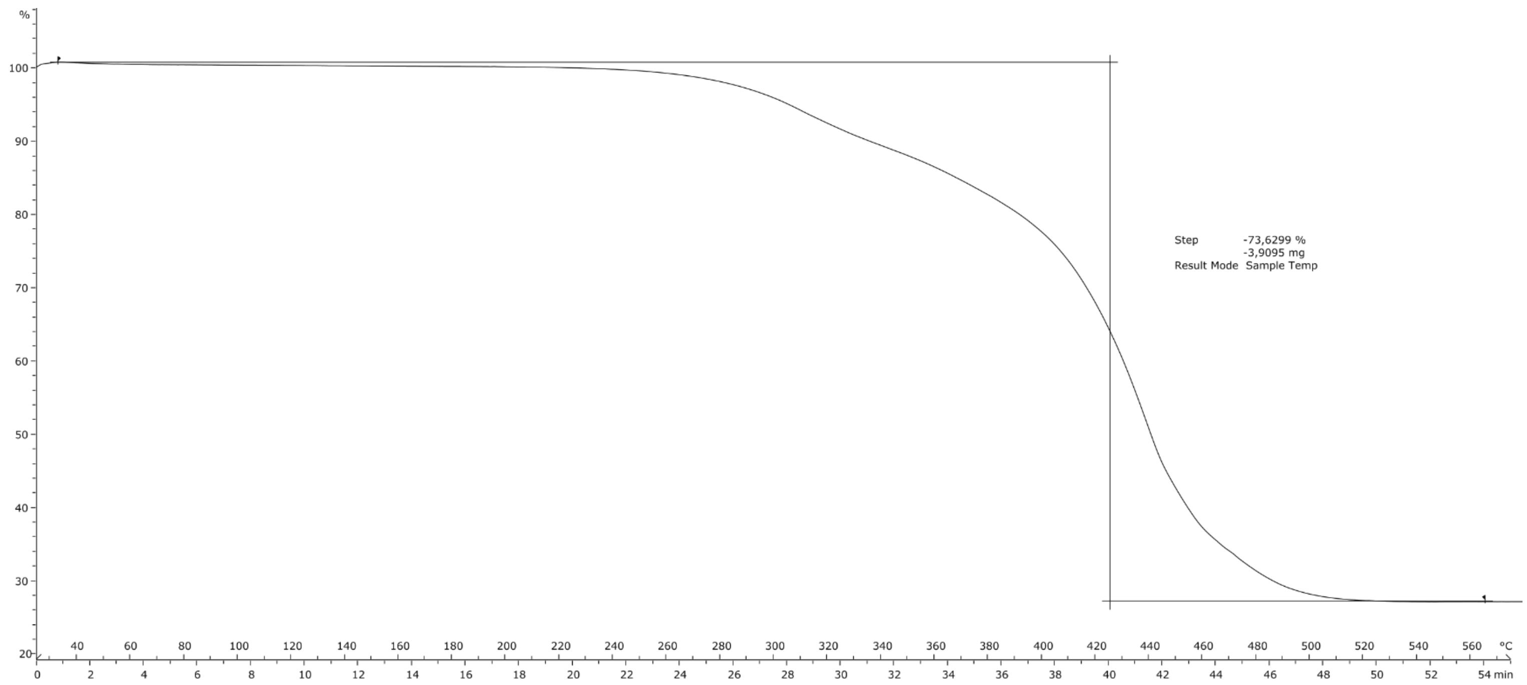

Figure S21. TGA curve of [Zn(PhCOO)$_2$]
